# Next-generation sequencing of representational difference analysis products for identification of genes involved in diosgenin biosynthesis in fenugreek (*Trigonella foenum*-*graecum*)

**DOI:** 10.1007/s00425-017-2657-0

**Published:** 2017-02-04

**Authors:** Joanna Ciura, Magdalena Szeliga, Michalina Grzesik, Mirosław Tyrka

**Affiliations:** Department of Biotechnology and Bioinformatics, Faculty of Chemistry, Rzeszow University of Technology, al. Powstańców Warszawy 6, 35-959 Rzeszów, Poland

**Keywords:** Diosgenin, Next-generation sequencing, Phytosterols, Representational difference analysis of cDNA, Steroidal saponins, Transcriptome user-friendly analysis

## Abstract

**Representational difference analysis of cDNA was performed and differential products were sequenced and annotated. Candidate genes involved in biosynthesis of diosgenin in fenugreek were identified. Detailed mechanism of diosgenin synthesis was proposed.**

Fenugreek (*Trigonella foenum*-*graecum* L.) is a valuable medicinal and crop plant. It belongs to Fabaceae family and has a unique potential to synthesize valuable steroidal saponins, e.g., diosgenin. Elicitation (methyl jasmonate) and precursor feeding (cholesterol and squalene) were used to enhance the content of sterols and steroidal sapogenins in in vitro grown plants for representational difference analysis of cDNA (cDNA-RDA). To identify candidate genes involved in diosgenin biosynthesis, differential, factor-specific libraries were subject to the next-generation sequencing. Approximately 9.9 million reads were obtained, trimmed, and assembled into 31,491 unigenes with an average length of 291 bp. Then, functional annotation and gene ontogeny enrichment analysis was performed by aligning all-unigenes with public databases. Within the transcripts related to sterol and steroidal saponin biosynthesis, we discovered novel candidate genes of diosgenin biosynthesis and validated their expression using quantitative RT-PCR analysis. Based on these findings, we supported the idea that diosgenin is biosynthesized from cycloartenol via cholesterol. This is the first report on the next-generation sequencing of cDNA-RDA products. Analysis of the transcriptomes enriched in low copy sequences contributed substantially to our understanding of the biochemical pathways of steroid synthesis in fenugreek.

**Electronic supplementary material:**

The online version of this article (doi:10.1007/s00425-017-2657-0) contains supplementary material, which is available to authorized users.

## Introduction

Fenugreek (*Trigonella foenum*-*graecum* L.) is an annual dicotyledonous legume belonging to the family of Fabaceae. Its genome size is 685 Mbp, i.e., approximately 1.5 × larger than the model legumes, *Lotus corniculatus* L. var. *japonicus* Regel and barrel (*Medicago truncatula* Gaertn.), both of which have compact genomes of about 470 Mbp (Vaidya et al. 2013). At present, fenugreek is widely cultivated in India, Argentina, Egypt, and Mediterranean countries. Besides culinary applications, fenugreek leaves and seeds are also consumed for medicinal purposes (Mehrafarin et al. 2011), as they show anti-diabetic, hypocholesterolemic, anti-microbial, and anti-cancer effects. This highly potent female herb helps relaxing the uterus and relieving menstrual pains, is an excellent stimulator of milk production in nursing mothers, and is usually suggested in treatments of poor digestion, gastric inflammations, enteritis, especially for convalescents (Petropoulos 2002; Mehrafarin et al. 2011; Patel et al. 2014).

Fenugreek plants contain active compounds such as alkaloids (e.g., trigonelline, gentianine, choline), flavonoids (e.g., apigenin, luteolin, quercetin, vitexin), steroids (e.g., cholesterol and sitosterol), saponins (e.g., diosgenin, gitogenin, tigogenin), lysine-rich proteins, and volatile oils (Snehlata and Payal 2012). Of these, the most important from pharmacological perspective is diosgenin—a steroidal saponin. Diosgenin is of great interest to pharmaceutical industry as it is used in the treatment of leukemia, hypercholesterolemia, climacteric syndrome, or colon cancer. Diosgenin is used as a substrate for synthesis of oral contraceptives, sex hormones, and other steroidal compounds (Patel et al. 2012). Its anti-cancer activity was proven, and it was found to contribute to an apoptosis of several cancer cell lines (i.e., colon HT-29 and HCT-116, breast AU565 and skin M4Beu) in vitro (Raju and Rao 2012).

Biosynthesis of steroidal saponins, including diosgenin, has not been described in detail yet. Glycolytic, mevalonate, and steroid biosynthesis pathways are involved in diosgenin biosynthesis, and cholesterol was found to be a precursor of this compound (Dewick 2002). It was established that cholesterol is formed from lanosterol and some of these reactions are catalyzed by cytochrome P450 systems (Mehrafarin et al. 2010). Vaidya et al. (2013) reported the first transcriptome sequencing of *T. foenum*-*graecum* and they concluded that diosgenin might be formed from squalene-2,3-oxide in two ways, (1) from lanosterol via the formation of cholesterol and (2) from cycloartenol via the formation of sitosterol.

The emergence of next-generation sequencing (NGS) has paved the way for large-scale sequencing of industrially important plants. At present, genomes of 39 model and non-model plants have been released and the data are publicly available (http://plants.ensembl.org/index.html). Transcriptomes of *Cicer arietinum* L., *Daucus carota* var. *sativus* L., *Hevea brasiliensis*, *Sesamum indicum* L., or *Camellia sinensis* have been sequenced recently (Annadurai et al. 2012). Sequences of transcriptomes are available also for 14 medicinal plant species (http://medicinalplantgenomics.msu.edu/index.shtml), and these data linked with metabolic profiles can be exploited to understand regulation and biosynthesis of selected target compounds important for medicinal properties. However, sequencing data for *T.* *foenum*-*graecum* are limited.

Efficient methods for profiling cDNA pattern changes in plants showing variable levels of target compounds are prerequisite for discovering new biosynthetic pathways. RNA-seq is a cost-effective, ultra-high-throughput DNA sequencing technology that provides a revolutionary advance in transcriptome-scale sequencing, and is competitive to a microarray technology. Direct sequencing of cDNAs generates short reads that can be assembled into a transcription profile. RNA-seq is a comprehensive and efficient way to qualitatively and quantitatively characterize transcriptomes, and discover new exons and splicing variants (Shi et al. 2011; Chikara et al. 2014). Significant number of medicinal plant species need to be profiled and analyzed for their transcriptomic data to help us (1) understand system biology of their metabolic pathways with reference to the metabolome, (2) compare them across the genes related to biogeneration of the same group of secondary metabolites and with the sets of genes with similar catalytic properties (gene families and super families), (3) elucidate metabolic pathways and their regulation, and (4) understand functional genomics of individual enzymatic step of significance/interest in the species (Sangwan et al. 2013).

There are a number of methods available for subtracting transcripts common for two pools of compared cDNAs. Although these methods are designed to determine qualitative differences between the two pools for up- and down-regulated genes, the subtraction should improve frequency of target genes. In this paper, we report on the first time use of NGS of representational difference analysis (RDA) products to discover the genes involved in diosgenin biosynthesis in fenugreek. We used the RDA-NGS approach to sequence the part of fenugreek transcriptome regulated by an elicitor (methyl jasmonate, MeJ) and precursors (cholesterol and squalene). The resulting assembled transcripts were functionally annotated, and the transcripts involved in the secondary metabolite biosynthesis pathways, especially of sterols and steroidal saponins, were analyzed. This study improved our understanding of molecular signatures in the transcriptome related to physiological functions of plant tissues associated with diosgenin biosynthesis pathway.

## Materials and methods

### Plant material

Seeds of *T. foenum*-*graecum* were obtained from the Botanic Garden in Bonn (Accession No. 19271). The plants were grown in vitro on Murashige and Skoog (1962) culture medium and were maintained at 25 °C with a 16-h light photoperiod. Six-month-old plants were sprayed with 10%-ethanol solutions of methyl jasmonate (MeJ) (100 µL L^−1^), squalene (100 µL L^−1^), and cholesterol (100 mg L^−1^). The leaves and stems elicited with MeJ or activated with squalene and cholesterol as precursors were harvested after 3 days, weighed, immediately frozen in liquid nitrogen and stored at −80 °C until further analysis. Control plants were sprayed with 10% solution of ethanol. The experiment was repeated three times. The tissues for analysis were crushed with mortar and pestle in liquid nitrogen. Homogenized samples were used for both RNA isolation and the diosgenin extraction.

### Diosgenin extraction and identification

Solid liquid extraction of diosgenin was performed as described by Savikin-Fodulovic et al. (1998), with modifications. The plant material was hydrolyzed with 1 M sulfuric acid in 70% isopropanol for 6 h under reflux. The next step included three cycles of extraction with hexane and then washing with 2 M NaOH and distilled water. Organic phase was evaporated to dryness on vacuum concentrator at 43 °C.

Diosgenin was identified by ultra performance liquid chromatography (UPLC) coupled with MS with triple quadrupole. A separation was run on C18 RP column (length of 50 mm, internal diameter of 2.1 mm, particle size of 1.7 μm). The isocratic solvent system was 85% methanol, flow rate was set at 0.6 mL min^−1^, and the column temperature was maintained at 25 °C. Retention time of diosgenin was 1.87 min. MS was equipped with an electrospray ion source operating in positive ion mode. For targeted metabolites, we used a very sensitive multiple reaction monitoring method. Transition for diosgenin *m*/*z* 415.3 → 271.2 and *m*/*z* 415.3 → 253.2 were detected. Measurements were taken in three replicates. Calibration curve was used to measure the concentration of diosgenin. Analysis of variances (*P* = 0.05) was employed to compare the concentrations of diosgenin.

### RNA extraction and cDNA library preparation

Total RNA from plants was isolated by GeneMATRIX Universal RNA Purification Kit (EURx, Gdansk, Poland). The quality and quantity of isolated total RNA were verified on 6000 Nano RNA chip and BioAnalyzer 2100 (Agilent Technologies). Samples with RNA integrity number above 7.5 were used in subsequent steps. Initially, 1 µg of total RNA was used for cDNA synthesis. ProtoScript II First Strand cDNA Synthesis Kit and mRNA Second Strand Synthesis Module (New England Biolabs) were used to obtain the first and the second strand of DNA.

### Representational difference analysis of cDNA (cDNA-RDA) and sequencing

To identify up-regulated genes in fenugreek in response to cholesterol, MeJ and squalene representational difference analysis of cDNA was performed. Samples with the highest diosgenin content obtained after treatment with cholesterol, MeJ, or squalene were the testers. Control samples with the lowest diosgenin level were used as drivers. Hubank and Schatz (1994) procedure was followed for cDNA-RDA, with small modifications. Three rounds of subtractive hybridization were carried out, and finally three differential, separate products resulting from the treatment with cholesterol, MeJ, and squalene were obtained. The third round yielded DNA bands and smear in the size range from 200 to 600 bp.

Transcriptome libraries for sequencing were constructed as outlined in Illumina’s Nextera XT DNA Library Preparation Guide. cDNA libraries were sequenced using paired end Illumina MiSeq. Raw data were saved in FASTQ format.

### De novo assembly and functional annotation

Bioinformatic analysis of raw reads was performed using Transcriptome User-Friendly Analysis (TRUFA; Kornobis et al. 2015), a webserver platform dedicated to RNA-seq analysis. A complete pipeline performs particular tasks in which read files are cleaned and then assembled into transcripts and finally the transcripts are identified and quantified. TRUFA executed the following steps (programs used are specified in the parentheses): reads quality control (FASTQC); quality trimming and duplicate removal (Prinseq); filtering out potential contaminants (Blat); de novo assembly of reads (Trinity); reads mapping (Bowtie2); contigs (i.e., transcripts) identification based on sequence alignment (Blat, Blast), protein dominions, profiles (HMMER) and annotation with gene ontology (GO) terms (Blast2GO); expression quantification.

For non-redundant protein database (NR) annotation, the Blast2GO PRO program (Conesa et al. 2005) was applied to obtain GO annotations according to molecular function, biological process, and cellular component ontologies. Each annotated sequence may have more than one GO term, either assigned in different GO categories (biological process, molecular function, and cellular component) or in the same category. Enzyme commission number was assigned and parsed based on the BLAST2GO results.

Additionally, unigenes (≥201 bp) were annotated by aligning against Swiss-Prot database by BLASTX (*e* value ≤ 1*e*−5) and EuKaryotic Orthologous Groups (KOG) databases using WebMGA server (Wu et al. 2011). WebMGA performs function annotation using RPSBLAST program on KOG database (eukaryotic proteins). Assembled sequences were also submitted to the online Kyoto Encyclopedia of Genes and Genomes database (KEGG) Automatic Annotation Server (KAAS) with bi-directional best hit method (Moriya et al. 2007).

### Validation of transcripts by qRT-PCR

Selected unigenes, annotated to sterol and sapogenin biosynthesis, were chosen for validation using real-time qPCR with gene-specific primers designed with Primer-Blast (Ye et al. 2012). One microgram of total RNA was converted to cDNA using M-MuLV (Moloney Murine Leukemia Virus) Reverse Transcriptase (EURx) by using oligo(dT) primers. Primers for the seven selected unigenes were designed (Table S1). The reaction was performed using GoTaq qPCR Master Mix kit (Promega) on Eco Real-Time PCR System (Illumina) using the following program: 95 °C for 10 min and 40 cycles of 95 °C for 15 s, anneal at 60 °C for 60 s.

The actin gene was selected as an internal standard for normalization, and three biological and two technical replicates were completed for each gene. Relative expression levels for each unigene were calculated using a delta–delta *C*_t_ ($2^{{ - \Delta \Delta C_{\text{t}} }}$) method. All data were expressed as the mean ± SD after normalization (Livak and Schmittgen 2001).

## Results

### Diosgenin content

Fenugreek plants were treated with methyl jasmonate (MeJ), cholesterol, and squalene to activate the genes involved in biosynthesis of diosgenin. Diosgenin content was determined in fresh plants after the treatments vs control (Fig. S1). Differences between samples were statistically significant. In respect to control (36.7 µg g^−1^), higher concentration of diosgenin in fresh plants was observed after treatment with MeJ (359 µg g^−1^), squalene (243 µg g^−1^), and cholesterol (186 µg g^−1^). Methyl jasmonate is a well-known compound that affects signal transduction involved in the elicitation process. The positive effect of MeJ on diosgenin production was previously reported (De and De 2011), and MeJ at 100 µL L^−1^ enhanced diosgenin content by 10.5 times. Cholesterol was applied for an induction of steroidal saponins (Aasim et al. 2014), but no information about the use of squalene was available.

### Sequencing and de novo assembly

Three final products of subtraction of cDNA pools (RDA-CHL, RDA-MeJ, and RDA-SQ) were sequenced on Ilumina MiSeq sequencer. Double-strand sequencing of *T.* *foenum*-*graecum* transcriptomes resulted in a total number of 9,918,886 reads (3,035,834 for RDA-CHL; 3,674,418 for RDA-MeJ; and 3,208,634 for RDA-SQ) with an average read length of 301 bp. The sequences were filtered by removal of duplicates and potential contaminants, and then quality-filtered sequences were submitted for *de novo* assembly. Cleaning and filtering reduced the number of sequences by about 33% to 1,036,467, 1,239,927, and 1,059,468 for RDA-CHL, RDA-MeJ, and RDA-SQ, respectively. De novo assembly was performed using Trinity (Grabherr et al. 2011) and reads were mapped through Bowtie2 (Langmead and Salzberg 2012) with default parameters implemented by TRUFA (Kornobis et al. 2015). A total of 11,603 contigs for RDA-CHL, 9029 for RDA-MeJ, and 10,859 contigs for RDA-SQ were generated (Table 1). An average length of contigs amounted to 290 bases, and only 2% of the sequences were longer than 500 bp. The transcripts were rich (about 57%) in AT nucleotides.

**Table 1:** Summary of sequences assembly

	RDA-CHL	RDA-MeJ	RDA-SQ
Total number of contigs	11,603	9029	10,859
Min length (bp)	201	201	201
Max length (bp)	843	730	743
Average length (bp)	293.30	287.94	292.91
Total bases in contigs (bp)	3,403,105	2,599,813	3,180,692
Number of contigs <500 bp	11,409	8935	10,673
Number of contigs ≥500 bp	194	94	186
N50 (bp)	295.0	289.0	295.0
Contigs in N50	4710	3730	4369
GC content (%)	42.46	43.45	42.55

### Functional annotation

Functional annotation of novel plant transcriptomes is a challenging task due to limited availability of reference genome/gene sequences in public databases (Annadurai et al. 2012). In non-model fenugreek plant, homologies with reference legumes (*L.* *corniculatus* and *M.* *truncatula*) are expected. Annotation of fenugreek transcriptome was based on sequence-based alignments. For this purpose, we performed a search against five public databases, including the NR, UniProt Reference Clusters (UniRef90), Swiss-Prot, KEGG, and KOG (Table 2). The highest number of hits (from 90.4% for RDA-CHL to 91.5% for RDA-MeJ) was found for NR and UniRef90 databases. Blast search against Swiss-Prot database revealed homologies for about 62% of unigenes.

**Table 2:** Summary of annotations of the *T. foenum*-*graecum* unigenes

	Number of sequences		
	RDA-CHL	RDA-MeJ	RDA-SQ
All assembled unigenes	11,720	9080	10,942
Gene annotations against NR	10,598	8316	9933
Gene annotations against UniRef90	10,467	8207	9827
Gene annotations against Swiss-Prot	7251	5754	6782
Gene annotations against KOG	5610	4583	5311
Gene annotations against KEGG	3123	2577	3001
Gene annotations against Pfam	9013	7104	8445

### Gene ontology (GO) classification

Based on NR annotations, GO analysis was performed to distribute unigenes into three major categories of cellular component, molecular function, and biological process. Annotated sequences may have variable number GO terms representing different categories. For all the unigenes selected in the RDA procedure, we found *Medicago truncatula*, *C. arietinum*, and *Glycine max* as the top-hit species with the highest distribution of similar sequences (Table S2). About 71% of transcripts within RDA pools were annotated with GO terms.

Distribution of GO terms between the three subtracted libraries was similar. Majority of the unigenes (53%) were annotated to biological processes, followed by molecular functions (27%) and cellular components (20%). In the biological processes category, the classes related to metabolic process (22%, GO:0008152), cellular process (20%, GO:0009987), single-organism process (17%, GO:0044699), biological regulation (7%, GO:0065007), and response to stimulus (6.7%, GO:0050896) were the most frequently observed. In molecular functions class, catalytic activity (45%, GO:0003824) and binding (42%, GO:0005488) were found to be the most abundant sub-categories. The most common GO terms within cellular components included a cell (36%, GO:0005623), organelle (26%, GO:0043226), and membrane (16%, GO:0016020).

### Multilevel GO analysis for biological process ontologies to trace secondary metabolism

The role of all the unigenes in secondary metabolism and sterol biosynthesis was analyzed using multilevel procedure (Table S3). At the third level, over 2300 of unigenes were involved in biosynthetic process and most of them played a role in the metabolism of small molecules and organic cyclic compounds. High number of sequences participated in the metabolic process of isoprenoid, terpenoid, and saponin biosynthesis. When compared to other RDA products, RDA-SQ transcripts were more abundant in steroid and sterol metabolic processes, and in biosynthesis of steroids, phytosterols, and sterols.

### Enzyme commission classification

**Fig. 1:**
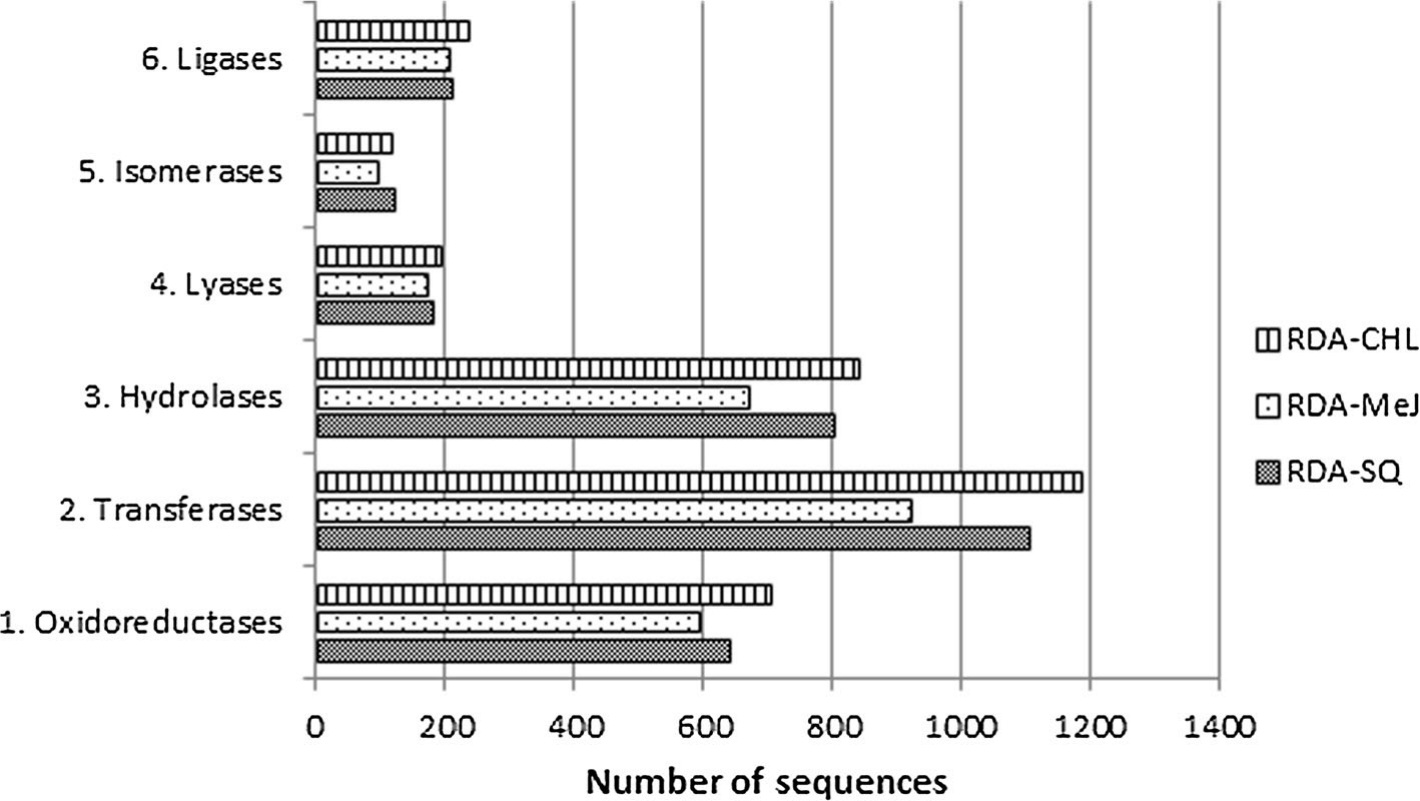
Enzyme commission classification of transcripts for different RDA pools

Depending on RDA pool, from 2667 to 3277 unigenes were classified as enzymes, and could be further divided into six subclasses. The most frequently represented enzymes were transferases (34.6–36.2%), hydrolases (25.2–26.2%), and oxidoreductases (20.9–22.3%). Ligases, lyases, and isomerases accounted for 3.6–7.8% of the enzymes, depending on RDA pool (Fig. 1).

The most frequently detected enzymes belonging to enzyme commission sub-categories for each RDA transcripts were 2.7, transferring phosphorus-containing groups (17.9% for RDA-CHL, 16.2% for RDA-MeJ, and 17.4% for RDA-SQ); 3.1, acting on ester bonds (7.1, 6.4, and 7.3% for RDA-CHL, RDA-MeJ, and RDA-SQ, respectively); 2.4, glycosyltransferases (6.2, 5.8, and 6.4% for RDA-CHL, RDA-MeJ, and RDA-SQ, respectively); 3.2, glycosylases (6.1, 6.2, and 6.2% for RDA-CHL, RDA-MeJ, and RDA-SQ, respectively); and 3.4, acting on peptide bonds (peptide hydrolases) (6.0, 6.2, 6.0% for RDA-CHL, RDA-MeJ, RDA-SQ, respectively).

### Kyoto Encyclopedia of Genes and Genomes (KEGG) pathway mapping

To identify the biological pathways that are active in fenugreek, all the assembled sequences were assigned with KEGG orthology (KO) identifiers using KEGG Automatic Annotation Server (KAAS) with the bi-directional best hit information method. We focused on steroid and terpenoid pathways to identify the enzymes involved in steroidal sapogenin biosynthesis. As a result of KEGG annotations, 3123, 2577, and 3001 sequences were assigned for RDA-CHL, RDA-MeJ, and RDA-SQ, respectively. In selected KEGG sub-categories, the most frequently represented pathways were those related to ribosome, spliceosome, protein processing in endoplasmic reticulum, RNA transport, starch and sucrose metabolism, and glycolysis/gluconeogenesis (Table S4).

Two hundred and sixteen unigenes in RDA-CHL, 187 in RDA-MeJ, and 210 unigenes in RDA-SQ were significantly matched to 26 KEGG pathways related to secondary metabolite biosynthesis (Table S5). We found 36, 35, and 43 unigenes encoding enzymes involved in steroid and terpenoid backbone biosynthesis in RDA-CHL, RDA-MeJ, and RDA-SQ libraries.

### Classification at Eukaryotic Orthologous Groups database (KOG)

The KOG proteins from the eukaryotic clusters were used to annotate 5610 transcripts for RDA-CHL (47.87% of all transcripts), 4583 for RDA-MeJ (50.47%), and 5311 for RDA-SQ (48.54%) libraries (Table S6). Among 25 KOG categories, the three largest clusters for each RDA transcripts were signal transduction mechanisms (717, 545, and 659 counts for RDA-CHL, RDA-MEJ, and RDA-SQ, respectively), posttranslational modification, protein turnover, chaperones (698 RDA-CHL, 613 RDA-MeJ, 704 RDA-SQ), and general function prediction only (697 RDA-CHL, 562 RDA-MeJ, 667 RDA-SQ). The categories of defense mechanisms, nuclear structure, extracellular structures, and cell motility were represented by the smallest groups of unigenes. The category of secondary metabolites biosynthesis, transport, and catabolism was represented by about 4% of genes corresponding to 261, 211, and 234 transcripts from RDA-CHL, RDA-MeJ, and RDA-SQ libraries, respectively. The most abundant sequences in this category were those of cytochrome P450 with a total 79 unigenes for RDA-CHL (30.2%), 66 unigenes for RDA-MeJ (31.3%), and 68 unigenes for RDA-SQ (29%). Additionally, for each RDA product, the most frequent sequences were found for iron/ascorbate family oxidoreductases, alcohol dehydrogenases, multidrug resistance-associated proteins/mitoxantrone resistance proteins, ABC superfamily, and pleiotropic drug resistance proteins (PDR1-15).

### Pfam annotations

Searching against the Pfam database using profile hidden Markov model methods resulted in annotations of 9013, 7104, and 8445 transcripts for RDA-CHL, RDA-MeJ, and RDA-SQ selected sequences, respectively. The aim of this approach was to identify similarities at domain level. The proteins that have little similarity at nucleotide sequence level might share conserved structural domains. Based on the frequency of the occurrence of transcripts in each Pfam domain, we ranked the Pfam domains/families and listed the fifteen top abundant domains/families (Table S7).

Among Pfam annotated sequences, protein kinase domain and its sub-class protein tyrosine kinase were the most frequently represented. These domains regulate a majority of cellular pathways (Zhang et al. 2015). Next most abundant sequences contained WD40 domain that is important for signal transduction mechanisms (Annadurai et al. 2012). The other frequently occurring domains in RDA products were cytochrome P450 (that mediates oxidation of organic substances and plays a significant role in secondary metabolite biosynthesis) and major facilitator superfamily (a class of membrane transport proteins that facilitate movement of small solutes across cell membranes). Other protein domains like RNA recognition motif, zinc finger, or leucine-rich repeats were also found.

### Identification of transcripts related to terpenoid backbone biosynthesis

Terpenoid backbone is formed from condensation of five-carbon building blocks designated as 3-isopentenyl pyrophosphate (IPP, C5) and dimethylallyl pyrophosphate (DMAPP, C5). In plants, IPP and DMAPP either derive from cytosolic mevalonate pathway via condensation of acetyl-CoA or from pyruvate and phosphoglyceraldehyde in the plastidial non-mevalonate pathway (MEP) (Augustin et al. 2011). In the next step, three isoprene units are linked to each other in a head-to-tail manner, resulting in a 15 C-atom molecule of farnesyl pyrophosphate (FPP). Two farnesyl pyrophosphates are subsequently linked in a tail-to-tail manner to give squalene, which is subsequently epoxygenated to 2,3-oxidosqualene. 2,3-oxidosqualene is considered a precursor of triterpenoid saponins, phytosterols, and steroidal saponins (Vincken et al. 2007; Xue et al. 2012). Two important pieces of information on this pathway are that mevalonate is not an intermediate in the plastidial synthesis of IPP and DMAPP, and that the MEP pathway appears to be largely responsible for the biosynthesis of monoterpenes, diterpenes, tetraterpenes (carotenoids), and polyprenols. Sesquiterpenes, sterols, and triterpenes are synthesized in the cytosol via the mevalonate pathway, which is responsible for the synthesis of the key intermediate FPP (Chappell 2002).

We found 34, 25, and 31 unigenes related to terpenoid backbone biosynthesis in RDA-CHL, RDA-MeJ, and RDA-SQ libraries, respectively (Table S8). Almost all of the genes encoding the enzymes involved in this pathway were present in our data. It was suggested that 3-hydroxy-3-methylglutaryl-CoA reductase (HMGR), squalene synthase (SQS), and squalene epoxidase (SQE) enzymes of the mevalonate pathway represented the rate-limiting or regulatory enzymes for saponin biosynthesis (Hwang et al. 2015). We identified 12, 5, and 4 non-redundant unigenes corresponding to HMGR, SQS, and SQE, respectively. Single or multiple unigenes were assigned to the enzymes involved in terpenoid backbone biosynthesis. These unique sequences could be assigned to different members of a gene family, different fragments of a selected gene, or both (Upadhyay et al. 2014).

### Identification of transcripts related to cholesterol biosynthesis and phytosterol pathway

Sterols are isoprenoid-derived molecules that have essential functions in eukaryotes in general, and in higher plants in particular. In vertebrates, cholesterol is by far the major sterol, whereas a mixture of various sterols is present in higher plants: sitosterol (usually predominant), stigmasterol, campesterol, and other. In plants, sterol biosynthesis pathway yields 24-methyl- and 24-ethyl-sterols. A number of minor sterols were detected suggesting that in addition to the main sterol biosynthesis pathway depicted here a number of side pathways existed (Benveniste 2004).

In the pathway including cholesterol, we identified Δ^14^-sterol reductase (EC:1.3.1.70), sterol-4α-methyl oxidase (EC:1.14.13.72), Δ^24^-sterol reductase (EC:1.3.1.72), cholestenol delta-isomerase (EC:5.3.3.5), and Δ^7^-sterol-C5(6)-desaturase (EC:1.14.21.6). For phytosterol biosynthesis via a sitosterol, we found cycloartenol synthase CAS (5.4.99.8), sterol 24-C-methyltransferase SMT1 (EC:2.1.1.41), cycloeucalenol cycloisomerase CPI (EC:5.5.1.9), sterol 14-demethylase CYP51 (EC:1.14.13.70), Δ^14^-sterol reductase FK (EC:1.3.1.70), Δ^8^-Δ^7^-sterol isomerase HYD1 (EC:5.3.3.5), 24-methylenesterol C-methyltransferase SMT2 (EC:2.1.1.143), sterol-4α-methyl oxidase SMO (EC:1.14.13.72), 3β-hydroxysteroid-4α-carboxylate 3-dehydrogenase HSD (1.1.1.–), Δ^7^-sterol-C5(6)-desaturase DWF7 (EC:1.14.21.6) 7-dehydrocholesterol reductase DWF5 (EC:1.3.1.21), Δ^24^-sterol reductase DWF1 (EC:1.3.1.72), and cytochrome CYP710A (Table S8 and S9).

### Validation of selected transcripts by qRT-PCR

To examine the expression pattern of genes encoding the enzymes involved in steroid biosynthesis pathways, we used qRT-PCR. Fragments per kilobase of exon per million fragments mapped (FPKM) values from expression quantification algorithm are the values used to measure relative expression of a transcript. In our study, the FPKM ranged from 0 to 4809.92, which indicated that unigenes showed a wide range of expression level (Table S9). We compared the values of FPKM and sequences of unigenes from RDA-CHL, RDA-MeJ, and RDA-SQ for individual enzymes involved in sterol and diosgenin biosynthesis. Seven unigenes were selected and RT-PCR was successfully applied to amplify unique products of the expected sizes. Based on the delta–delta *C*_t_ ($2^{{ - \Delta \Delta C_{\text{t}} }}$) method, relative expression levels of the selected unigenes were calculated and compared among the three differential products.

**Fig. 2:**
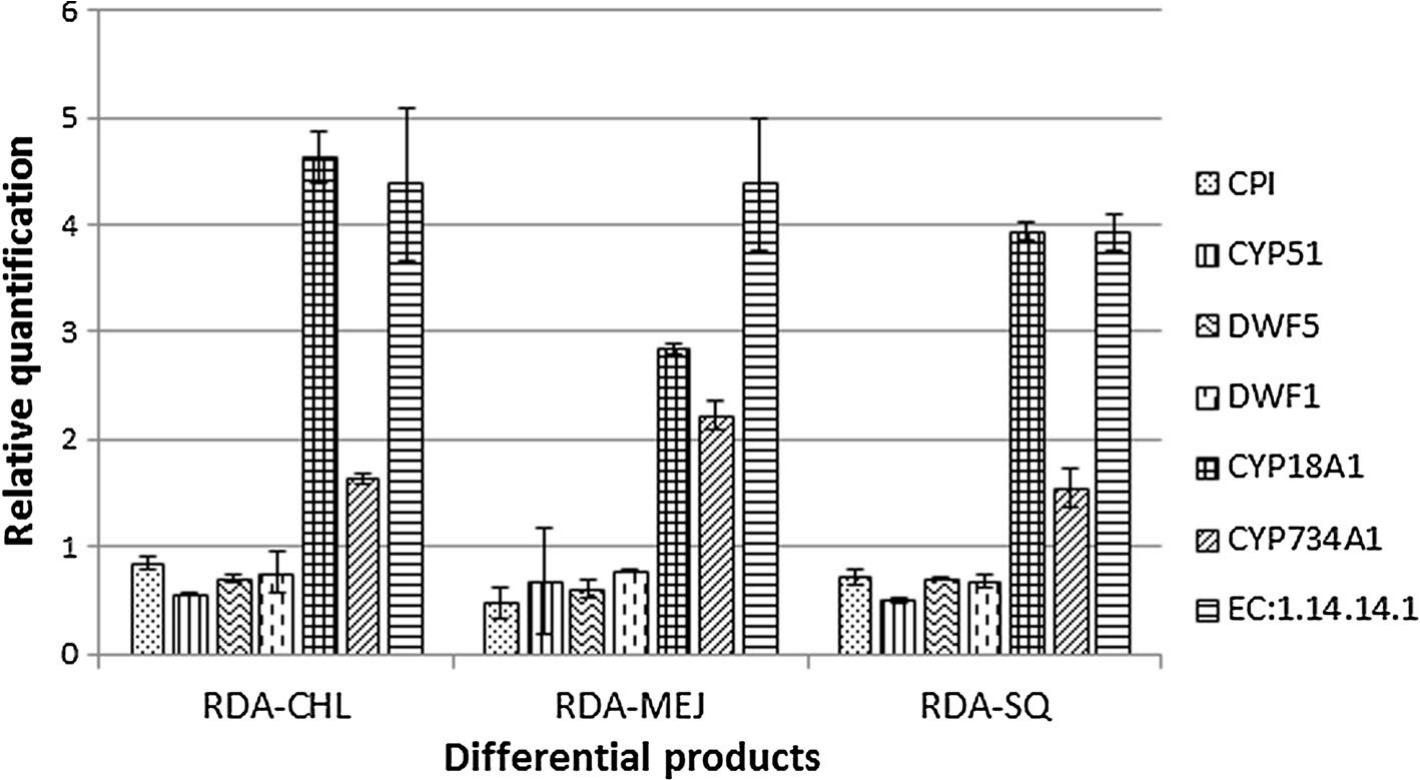
Relative expression levels of secondary metabolism-related gene transcripts. *Vertical bars* denote ± SD. *CPI* cycloeucalenol cycloisomerase, *CYP51* sterol 14-demethylase, *DWF5* 7-dehydrocholesterol reductase, *DWF1* sterol Δ24-reductase, *CYP18A1* 26-hydroxylase, *CYP734A1* 26-hydroxylase, *EC:1.14.14.1* unspecific monooxygenase

All the transcripts showed minor differences in relative quantification RQ (Fig. 2). Increased expression was identified for CYP18A1, CYP734A1, and unspecific monooxygenase. The greatest differences between cDNA-RDA products were determined for CYP18A1 and unspecific monooxygenase EC:1.14.14.1.

**Fig. 3:**
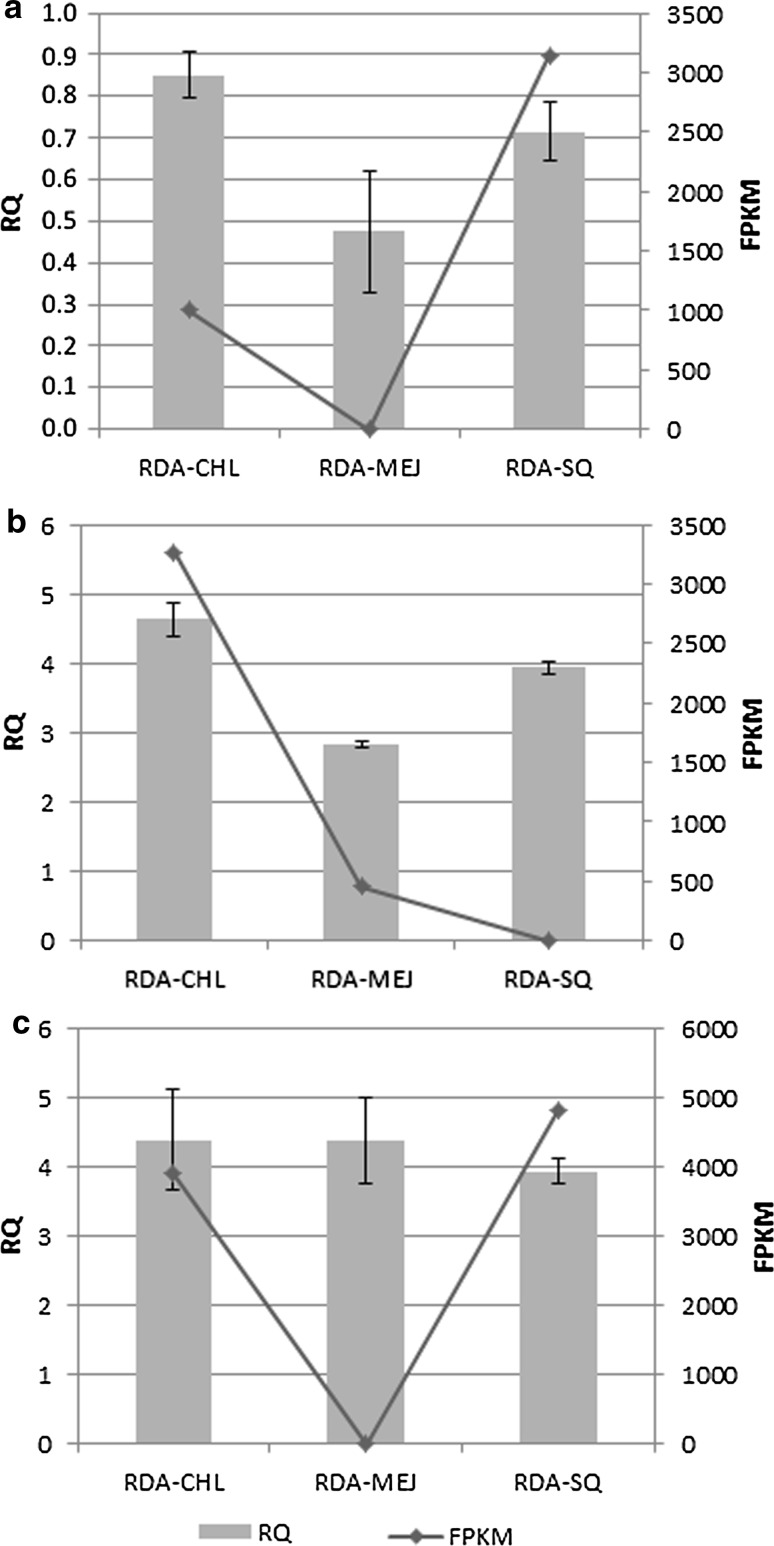
Quantitative qRT-PCR validations of cycloeucalenol cycloisomerase (**a**), CYP18A1 (**b**), and unspecific monooxygenase (**c**)

For three selected unigenes, high FPKM values were not supported by RQ scores (Fig. 3). For CPI FPKM, the RQ values were the highest for RDA-SQ and RDA-CHL, respectively. Similar results were obtained for unspecific monooxygenase. For CYP18A1, the highest FPKM was related with RDA-CHL and similar value was found for RQ.

Overall, the results of qRT-PCR experiments were in agreement with FPKM data, and confirmed that the selected unigenes were efficiently induced in cholesterol-, MeJ-, and squalene-treated samples, and that sequencing data obtained from the assembled transcriptome were reliable.

## Discussion

### Induction of diosgenin biosynthesis in vitro

Synthesis of secondary metabolites is often stimulated by biotic or abiotic stresses. Saponins exhibit a wide range of biological activities and take part in mechanisms of defense and protection as well as in environmental interactions (Szakiel et al. 2011). Saponins are used to defend plant against pathogens. Upon a pathogen infection, saponin content increases as a result of chemical modifications of their precursor molecules. Another role of saponins in plants is to protect them against herbivores and/or insects and this is mostly based on their acting as deterrents, toxins, and digestibility inhibitors. This is associated with their capacity to disrupt cell membranes (Faizal and Geelen 2013).

Plant cell and tissue culture systems represent a potential source of valuable secondary metabolites. A production in in vitro cultures is not dependent on geography and season, product quality and yield are generally established, and clonal propagation methods can be used to overcome germination and plant heterogeneity issues. In addition, improved production of desired compounds is easily achievable in in vitro cultures using biotechnological methods (Lambert et al. 2011). Plant cells in vitro show physiological and morphological responses to microbial, physical, or chemical factors that are known as elicitors. Elicitation is an induced or enhanced biosynthesis of metabolites triggered by an addition of trace amounts of elicitors (Smetanska 2008). These defense responses can be activated through a signal transduction pathway via recognition of an “elicitor” by specific receptors located in the plasma membrane and formation of secondary messengers, such as jasmonates, ethylene, and salicylic acid, which in turn activate the expression of defense genes, including the genes that encode the enzymes catalyzing the formation of secondary metabolites (Lambert et al. 2011). Numerous examples of elicitors that enhance saponin production can be found in research literature.

Upadhyay et al. (2014) studied transcriptional changes in leaf and root tissues of *Asparagus racemosus* after methyl jasmonate treatment. They found that the transcripts of UGTs and CYP450 involved in glycosylation and oxygenation steps were accumulated following the treatment. This up-regulation might be due to the fact that mono-oxygenases that catalyze oxygenation reactions and glycosyltransferases that catalyze the transfer of sugar molecules to steroidal compounds may produce diverse saponins under different conditions (stress) and control plant activities. Similarly, in *Eleutherococcus senticosus*, siberian ginseng, which was exposed to MeJ, the genes involved in saponin biosynthesis pathway that might be involved in triterpene formation, hydroxylation, or oxidation of triterpene skeletons by CYP and glycosylation by UGT were up-regulated (Hwang et al. 2015). Recently, Cao et al. (2015) found that MeJ treatment up-regulated squalene synthase, squalene epoxidase, and dammarenediol synthase in *Panax ginseng*. Also in fenugreek, the application of MeJ caused an increase in diosgenin level and enhanced the expression of 3-hydroxy-3-methylglutaryl-CoA reductase (HMGR) and sterol-3-glucosyltransferase genes involved in the biosynthetic pathway of saponins (Chaudhary et al. 2015). Application of methyl jasmonate induced the genes involved in saponin biosynthesis and accumulation. Our results confirmed the effectiveness of MeJ treatment in enhancing the production of diosgenin in fenugreek tissues.

### Next-generation sequencing of cDNA-RDA products

Representational difference analysis is a powerful and sensitive tool for the identification of differentially expressed genes in two different cDNA populations and it allows for detection of changes in mRNA expression by selective enrichment without any prior knowledge of the gene in question (Leite et al. 2013). Moreover, cDNA-RDA is capable of detecting both relative (greater than three- to five-fold) and absolute differences in expression (Hubank and Shatz 1999). This very high sensitivity and specificity of cDNA-RDA approach should allow the genes switched on, or up- or down-regulated, in both the treated sample and its control, to be analyzed in the same experiment (Bowler et al. 1999). In our study, comparison of samples with contrasting levels of diosgenin content made it possible to identify the genes involved in steroidal saponin biosynthesis. Application of RDA procedure to reduce the amount of overrepresented transcripts partially compensates lower sequencing resolution of Miseq when compared to Hiseq.

In this study, we sequenced RDA products and obtained a total of 31,491 unigenes (11,603 for RDA-CHL; 9029 for RDA-MeJ; and 10,859 for RDA-SQ) by de novo assembly. These unigenes were then annotated in different databases under various criteria like gene or protein name descriptions, conserved domains, GO terms, and metabolic pathways. Detailed functional information is very important to understand overall expression profiles and known biosynthetic pathways of secondary metabolites. The most annotated unigenes were found in NR and UniProt Reference Clusters 90 databases.

Methyl jasmonate stimulates defense reaction of plants to biotic and abiotic stresses. Cholesterol and squalene are expected to participate mainly in metabolism of terpenoids and sterols. Therefore, more unigenes can be expected after MeJ treatment, compared to cholesterol or squalene. However, sequencing of differential products revealed less unigenes after MeJ treatment than following the treatments with cholesterol and squalene (Tables S2–S5). The quantities of normalized cDNA libraries used for sequencing were equimolar and the yields of raw data were similar. If the targeted genetic change does not result in a unique DNA fragment after digestion, then the change cannot be detected (Chang 2003). Thus, the discrepancies in the number of unigenes can be explained by the nature of RDA procedure that equilibrates the levels of overexpressed transcripts and favors diversity of cDNA fragments present at low levels.

We found that four down-regulated genes (CPI, CYP51, DWF5, and DWF1) belonged to the cycloartenol to cholesterol pathway, and three up-regulated transcripts (CYP18A1, CYP734A1, and EC:1.14.14.1) represented cholesterol to diosgenin pathway. Activity of the last three genes may be directly connected with elevated levels of diosgenin (Fig. S1). This observation suggests that both pathways (leading to cholesterol and diosgenin) are under different regulatory mechanisms. Cholesterol is involved in a number of biological processes (including membrane permeability) and other regulatory mechanism can overlap to maintain homeostasis.

Several studies that exploited transcriptome sequencing can be referred to diosgenin biosynthesis. Sequencing of fenugreek and *Asparagus racemosus* transcriptomes (Vaidya et al. 2013; Upadhyay et al. 2014) suggested that saponins were formed from sitosterol via cycloartenol. In asparagus, *CYP51* and *DWF1* genes were overexpressed after MeJ treatment. Five genes (*SQE, CAS, CYP51, DWF5,* and *DWF1*) involved in the biosynthesis of phytosterols were found in *Dioscorea composite* (Wang et al. 2015). These genes were overexpressed in the leaves and tubers as compared with the stem and shoot. However, the relative level of expression exceeded the value of 1 only for CAS and CYP51 genes.

### Identification and expression analysis of candidate genes involved in steroidal sapogenin biosynthesis

Steroid pathway is the last step of steroidal sapogenin biosynthesis. There are two ways of diosgenin biosynthesis from 2,3-oxidosqualene: the first starts from lanosterol via the formation of cholesterol and the second starts from cycloartenol via sitosterol (Vaidya et al. 2013; Upadhyay et al. 2014). Phytosterols are synthesized from cycloartenol; however, the steps at which the biosynthesis of steroidal saponins and phytosterol diverges have not been elucidated (Faizal and Geelen 2013). The transcriptomes annotated within our experiment revealed multiple transcripts encoding all known enzymes involved in the steroid biosynthesis pathway. Based on the KEGG results, we found 10, 11, and 15 unigenes involved in steroid biosynthesis in RDA-CHL, RDA-MeJ, and RDA-SQ libraries, respectively. However, based on NR annotations, a number of unigenes were identified as “unknown,” “uncharacterized,” or “hypothetical protein,” although they possessed suitable domains, i.e., PF00432.16 (prenyltransferases and squalene oxidases) or PF01073.14 (3β hydroxysteroid dehydrogenases), that can be accounted to database of unigenes potentially involved in sterol biosynthesis. Some of these transcripts showed increased FPKM values, thus indicating enhanced expression. We annotated unigenes involved both in biosynthesis via a cholesterol and sitosterol, but at the similarities above 60% no transcripts corresponding to lanosterol synthase (EC:5.4.99.7) and 3-ketosteroid reductase (KR; EC:1.1.1.270) were found in RDA products (Table S8).

We identified unigene comp20077 (RDA-CHL) annotated as cytochrome P450 90B1-like, and contribution of this enzyme to steroidal sapogenin synthesis pathway can be considered. In brassinosteroid biosynthesis, C-22 steroid hydroxylase (CYP90B1) catalyzes hydroxylation of C-22. Furthermore, overexpression of CYP90B1 in *Escherichia coli* showed broad catalytic activity with respect to the sterols containing 27- (cholesterol, cholestanol), 28- (campesterol, campestanol), and 29-carbon atoms (sitosterol). CYP90B1 preferred plant sterols with carbon atoms C5-C6 having a double bond to stanols that contained no double bond. Recombinant CYP90B1 showed the highest catalytic activity in hydroxylation of cholesterol to 22-hydroxycholesterol (Fujita et al. 2006).

As regards diosgenin biosynthesis process, we hypothesized that ring E closure was created as a result of hemiketal reaction with –OH group in C-16 and =O group in C-22. The keto group may be formed by oxidized hydroxyl group. We supposed that oxidation in C-16 might be catalyzed by unspecific monooxygenase EC:1.14.14.1. This enzyme takes part in a number of reactions, i.e., oxidation of estrone to 16-hydroxyestrone; estradiol-17β to estriol; dehydroepiandrosterone to 16α-hydroxydehydroepiandrosterone. We found six unigenes for RDA-CHL, seven unigenes for RDA-MeJ, and six unigenes for RDA-SQ that were annotated as cytochrome p450 EC:1.14.14.1 (Table S9). Especially for RDA-SQ, four of the six unigenes had high values of FPKM (from 329.80 to 4809.92).

The next stages of brassinosteroid biosynthesis involve cytochrome CYP734A1 with 26-hydroxylase activity that converts brassinolide and castasterone into their 26-hydroxy derivatives (Turk et al. 2003). We annotated four unigenes for RDA-CHL (comp3187, comp4513, comp51591, comp73533), two unigenes for RDA-MeJ (comp2112, comp2587), and four unigenes for RDA-SQ (comp101982, comp3589, comp4033, comp51388). These unigenes could also be involved in steroidal sapogenin biosynthesis. Some of them had raised FPKM values (comp4513 1241.42; comp3589 1107.27; comp4033 785.41).

Within differentially expressed transcripts, we annotated also cytochrome P450 with 26-hydroxylase activity orthologous with CYP18A family (KEGG K14985) that can be involved in diosgenin biosynthesis. CYP18A1 enzyme takes part in insect hormone biosynthesis, catalyzes hydroxylation of 20-hydroxyecdysone to 20,26-dihydroxyecdysone, and is a key enzyme of *Drosophila* steroid hormone inactivation. We found two corresponding unigenes, comp6079 (RDA-CHL) and comp2698 (RDA-MeJ) with high FPKM values (3264.61 for comp6079 and 453.18 for comp2698), which confirmed overexpression of these transcripts and suggested the role of the corresponding enzyme in hydroxylation of C-26.

Ecdysteroids, present mainly in insects and other invertebrates, were found also in many plant species (more than 300 different phytoecdysteroids). Although ecdysteroids originate from sterols, their biosynthesis and intermediates have not been established yet. For example, in fern 22- and 25-hydroxy derivatives of cholesterol were converted to ecdysone and 22-hydroxyecdysone (Dinan et al. 2009). The presence of ecdysteroids in plants and overexpression of unigenes orthologous to CYP18A in the analyzed samples led to the conclusion that this cytochrome might recognize a structure of the sterol and participate in the biosynthesis of diosgenin.

Further enzymes and reactions resulting in diosgenin biosynthesis are unknown but cytochromes may play a significant role. After oxidation and hydroxylation of carbon C16, C22, and C26 and creation of a hemiketal form, the next step is to create the F ring by converting furostan form (aliphatic chain) into spirostan (ring F). Closing aliphatic chain to form ring F in spirostan requires both 26-*O*-glucoside and –OH group in C-22. An addition of glucose molecule to a hydroxyl group in C-26 (glycosylation) is probably catalyzed by sterol glucosyltransferase. Glucose is transferred from uridine diphosphate glucose (UDP-glucose); therefore, the enzyme is UDP-glucosyltransferase (2.4.1.–) (Kalinowska et al. 2005). Furostanol saponins are usually present as 26-*O*-glucosides and activity of specific 26-*O*-β-glucosidases that eliminate glucose results in the creation of F ring (Moreau et al. 2002). Protodioscin 26-*O*-β-d-glucosidase (EC:3.2.1.186) that catalyzes a cleavage of glucose from protodioscin and formation of 26-deglucoprotodioscin was characterized in *Solanum torvum*. In fenugreek transcripts selected upon MeJ treatment, we found two unigenes showing high similarity to protodioscin 26-*O*-β-d-glucosidase at protein level. Sequences comp17664 (alignment 33% *e* value 6*e*−09) and comp91232 (52% alignment *e* value 7*e*−07) were annotated as β-glucosidases (EC:3.2.1.21).

### Mechanism of diosgenin biosynthesis from cycloartenol via cholesterol

The annotated unigenes were used to study a secondary metabolic pathway. We focused on the genes and enzymes that could be involved in steroidal sapogenin biosynthesis. Terpenoid backbone is synthesized from mevalonate pathway leading to 2,3-oxidosqualene. Our results suggested that cholesterol was synthesized from cycloartenol. Diener et al. (2000) proposed a pathway leading from cycloartenol to cholesterol and its 24-methyl and 24-ethyl derivatives. We proposed a similar pathway, where double bond in C24 was maintained to the stage when desmosterol was reduced to cholesterol. Biosynthesis of cholesterol from cycloartenol in plants was also recently proposed by Talapatra and Talapatra (2015). Our hypothesis was further confirmed by a lack of transcripts corresponding to lanosterol synthase—an enzyme essential in this biosynthesis beginning from lanosterol in animals and fungi.

Cycloartenol synthase (CAS) and lanosterol synthase have similar (77–79%) amino acid sequences in *Arabidopsis thaliana* (Suzuki et al. 2006; Umate 2015). In sequenced subtraction libraries, only two unigenes (comp99 and comp166794) were annotated as CAS and no sequence corresponding to lanosterol synthase was identified. The two sequences comp99 and comp166794 were aligned using blastx and showed 85 and 98% identity to cycloartenol synthase from *Arabidopsis thaliana* and *Medicago truncatula*, respectively. We also found 60% homology of these sequences to lanosterol synthase from *A. thaliana* (*LAS1*, At3g45130), while no gene annotated as lanosterol synthase was identified for *M. truncatula*.

**Fig. 4:**
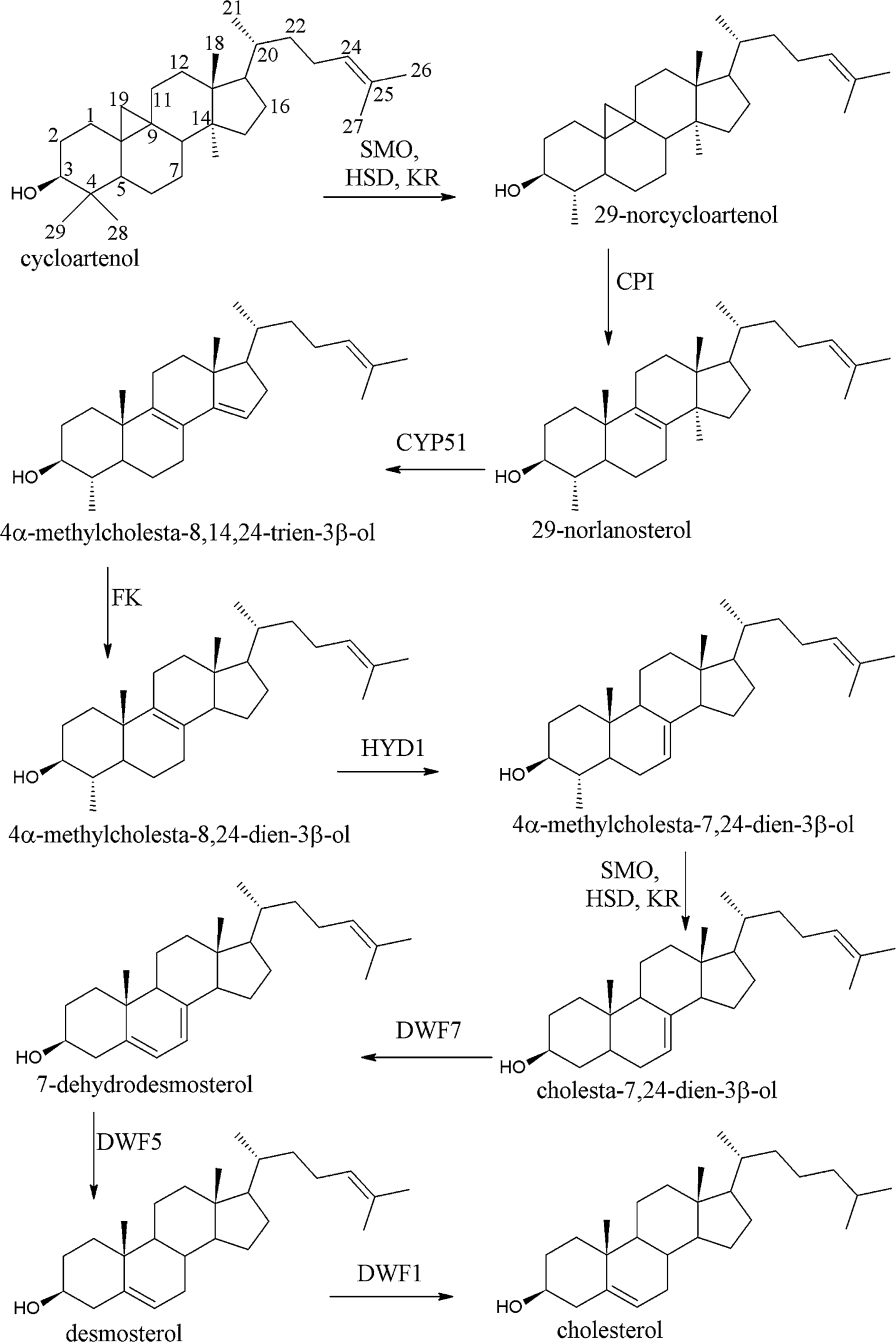
Proposed pathway leading from cycloartenol to cholesterol in steroidal sapogenin biosynthesis. *SMO* sterol-4α-methyl oxidase (EC:1.14.13.72), comp60361 (XP_006358705.1), *HSD* 3β-hydroxysteroid-4α-carboxylate 3-dehydrogenase comp456, comp147036 (XP_003603241.1), comp27806 (XP_004501411.1), *KR* 3-ketosteroid reductase, *CPI* cycloeucalenol cycloisomerase (EC:5.5.1.9) comp1007, comp3693, comp50967 (XP_003602088.1), comp230 (XP_004500654.1), *CYP51* sterol 14-demethylase (EC:1.14.13.70) comp4337, comp2760, comp4118 (ABC59074.1), *FK* Δ14-sterol reductase (EC:1.3.1.70) comp3967, comp21207, comp2560 (XP_004497600.1), *HYD1* Δ^8^-Δ^7^-sterol isomerase (EC:5.3.3.5) comp71327 (XP_003534176.2), *DWF7* Δ^7^-sterol-C5-desaturase (EC:1.14.21.6) comp60818 (XP_003849939.1), *DWF5* 7-dehydrocholesterol reductase/sterol Δ^7^-reductase (EC:1.3.1.21) comp4188, comp3298, comp2096, comp3395, (XP_004507556.1), *DWF1* sterol Δ^24^-reductase (EC:1.3.1.72) comp4546, comp2786, comp3715 (P93472.1)

We propose a pathway of cholesterol biosynthesis (Fig. 4) that involves the same enzymes that catalyze phytosterol biosynthesis, except for sterol methyltransferases SMT1 and SMT2. Cycloartenol is formed from 2,3-oxidosqualene by cycloartenol synthase (5.4.99.8) and the next step—methylation of C-24 is skipped. In cycloartenol, one methyl group from C-4 is removed by sterol-4α-methyl oxidase (SMO), 3-β-hydroxysteroid-4α-carboxylate 3-dehydrogenase (HSD), and 3-ketosteroid reductase (KR). Then, CPI isomerizes the cyclopropane ring in 29-norcycloartenol to form Δ^8^ tetracycles present in the tetracyclic sterols. The next step is demethylation of C-14 and reduction of the resulting bond by CYP51 and FK, respectively. CYP51, a member of cytochrome P450 monooxygenase superfamily, is essential for sterol biosynthesis and it is assumed to be the only orthologous P450 family that exists in the fungi, mammal, and plant kingdom. HYD1 catalyzes a removal of a hydrogen from 7β position and subsequent addition of a hydrogen at 9α position, reducing the existing Δ8(9) double bond and producing Δ7(8) double bond of the sterol B ring. Then, second demethylation step from C-4 is catalyzed by SMO, HSD, and KR. DWF7 is involved in Δ^7^ sterol C-5 desaturation step and DWF5 reduces the double bond in C-7. The last stage is a reduction of Δ^24^-bond of sterols and formation of cholesterol by DWF1.

The proposed pathway retains the order of changes occurring in the biosynthesis of phytosterols: methyl groups are removed in the order of C4, C14, and C4, and there is a close cooperation of three enzymes (SMO, HSD, and KR) catalyzing two reactions of demethylation from C4 (Rahier 2011) and sequential reactions of C14 reduction, and the double bond isomerization of C8 to C7 (Schrick et al. 2002).

**Fig. 5:**
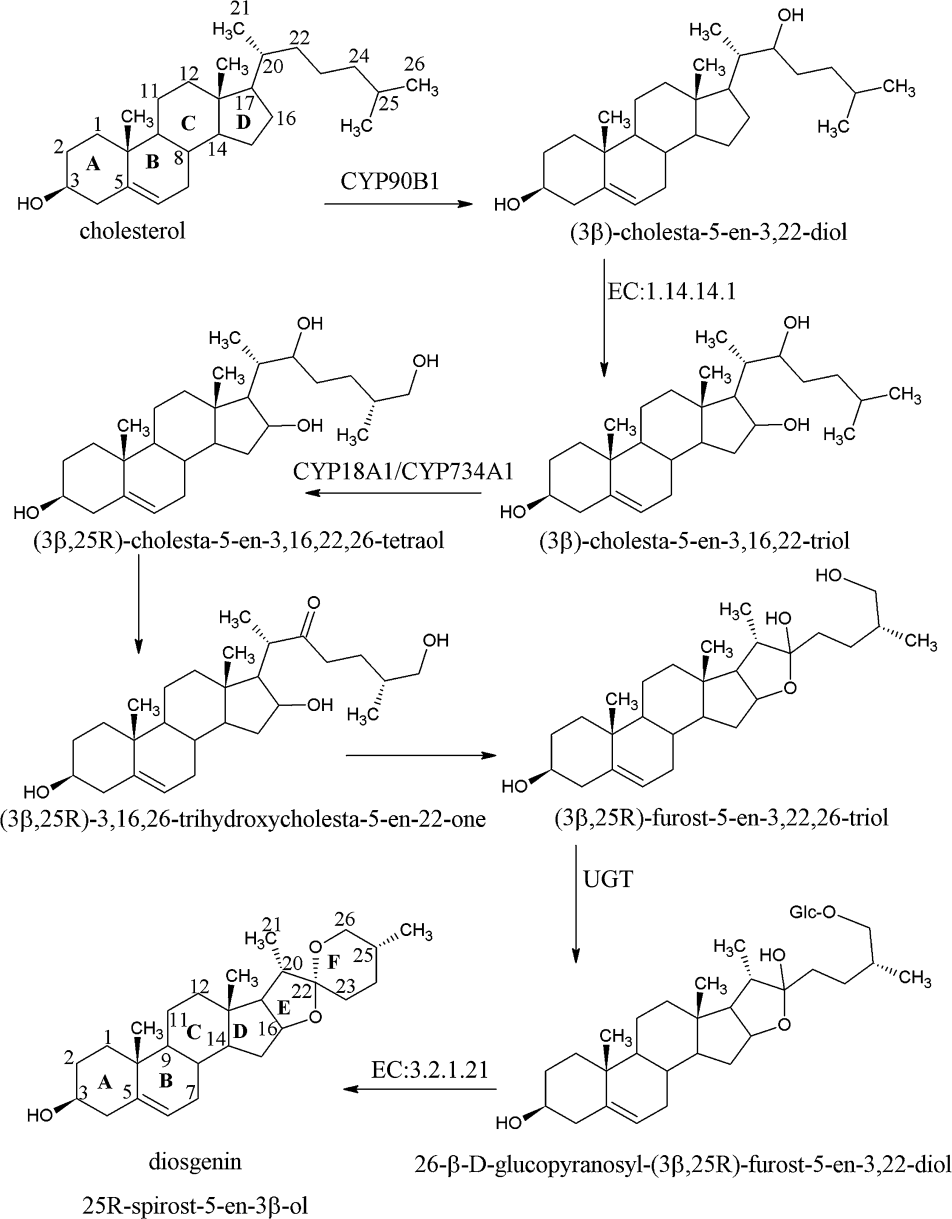
Proposed pathway leading form cholesterol to diosgenin. *CYP90B1* steroid 22α-hydroxylase comp20077 (XP_003629467.1), *EC:1.14.14.1* unspecific monooxygenase comp35839, comp88342, comp32891 (XP_003610974.1), *CYP18A1/CYP734A1* cytochromes that catalyze C-26 hydroxylation comp6079 (XP_003615674.1), comp2698 (XP_007036488.1), comp4513, comp2112, comp3589 (XP_004495852.1), *UGT* UDP-glucosyltransferase comp2754 (XP_008085517.1), *EC:3.2.1.21* β-glucosidase comp1334, comp17664, comp2749 (XP_006575592.1)

For the first time, we proposed the enzymes that are involved in further processing of cholesterol (Fig. 5). We propose that in this process unspecific monooxygenase EC:1.14.14.1 participates in hydroxylation of C-16, and CYP18A1/CYP734A1 catalyzes hydroxylation of C-26 and probably precedes the reaction of glucose addition to cholesterol. Moreover, qRT-PCR data suggest that hydroxylation of C-22 may be catalyzed by steroid 22α-hydroxylase (CYP90B1). The last two stages involve the closure of the F ring and formation of spiroketal. First, the glycosylation of C26 is catalyzed by UDP-glucosyltransferase, and then glucose molecule is removed by β-glucosidase (EC:3.2.1.21) to form a spiro ring of diosgenin. The order of carbon hydroxylation of C16, C22, and C26 needs to be confirmed experimentally. Alternative pathways of diosgenin biosynthesis in fenugreek induced by inhibitors or resulting from genetic diversity may also be feasible.

## Conclusions

We reported on the application of next-generation sequencing (NGS) technology for the analysis of cDNA-RDA products after treatment with methyl jasmonate, cholesterol, and squalene. Subtraction procedure equilibrated the frequencies of transcripts and increased a chance to identify unique genes. De novo assembly and functional annotations were performed. As a result of the analysis of related pathways, we identified the majority of candidate genes involved in important secondary metabolic pathways as sterols and steroidal sapogenins. Moreover, we propose a biosynthetic pathway leading to formation of diosgenin from cycloartenol via cholesterol.

### *Author contribution statement*

JC collected the literature, prepared plant material, performed UPLC analysis, participated in RNA isolation, RDA procedure and sequencing, interpreted scientific information, and wrote the manuscript. MS participated in RNA isolation and RDA procedure and helped in data interpretation. MG participated in RDA procedure, library preparation, and sequencing. MT coordinated the experiment, was involved in scientific advising, and helped in the manuscript edition.

## Electronic supplementary material

Below is the link to the electronic supplementary material.

Supplementary material 1 (DOCX 13 kb)

**Fig. S1** Average content of diosgenin in fenugreek plants (PNG 12 kb)

Supplementary material 3 (DOCX 12 kb)

Supplementary material 4 (DOCX 13 kb)

Supplementary material 5 (DOCX 26 kb)

Supplementary material 6 (DOCX 14 kb)

Supplementary material 7 (DOCX 14 kb)

Supplementary material 8 (DOCX 13 kb)

Supplementary material 9 (DOCX 17 kb)

Supplementary material 10 (XLSX 38 kb)

## Abbreviations

cDNA-RDA: Representational difference analysis of cDNA
FPKM: Fragments per kilobase of exon per million fragments mapped
GO: Gene ontology
KEGG: Kyoto Encyclopedia of Genes and Genomes database
KOG: Eukaryotic orthologous groups database
MeJ: Methyl jasmonate
NR: Non-redundant database
RDA-CHL: Differential product after cholesterol treatment
RDA-MeJ: Differential product after methyl jasmonate treatment
RDA-SQ: Differential product after squalene treatment
