# Supplementary material for: Next-generation sequencing of representational difference analysis products for identification of genes involved in diosgenin biosynthesis in fenugreek (Trigonella foenum-graecum)
Source: Planta. 2017 Feb 4;245(5):977–91. doi: 10.1007/s00425-017-2657-0 (PMC5393294; doi:10.1007/s00425-017-2657-0)
Supplement: Supplementary file 1 — Supplementary material 1 (DOCX 13 kb) [file 425_2017_2657_MOESM1_ESM.docx]

Next generation sequencing of representational difference analysis products for identification of genes involved in diosgenin biosynthesis in fenugreek (*Trigonella foenum-graecum*), Planta, Ciura J, Szeliga M, Grzesik M, Tyrka M; Department of Biotechnology and Bioinformatics, Rzeszow University of Technology, Poland, mtyrka@prz.edu.pl

Table S1 Primers used for determination of the levels of selected transcripts with qRT-PCR

| Enzyme name | Primer sequences 5' to 3' |
| --- | --- |
| Cycloeucalenol cycloisomerase (CPI) | F: CAGAAATTTGGCACTGCAAGC |
|  | R: GGGTCCAATTGTTCCGGTCT |
| Sterol 14-demethylase (CYP51) | F: ATACCTGCCAATCCCAGCTC |
|  | R: TGTGCTGTCCTGCGAAAAGA |
| 7-dehydrocholesterol reductase (DWF5) | F: GTGCAAGGACAGCCCATGA |
|  | R: TCAACACTCATCTTTGGGAGC |
| Delta(24)-sterol reductase (DWF1) | F: GGTGTTTGATGGCGTGGAAG |
|  | R: TCCTTCTCGCTTTTCCTGCC |
| 26-hydroxylase (CYP18A1) | F: AAGGGCGTAGGAAAGGAACA |
|  | R: CTGCTTTGGTGGGTGAGGTT |
| 26-hydroxylase (CYP734A1) | F: ATGGGAAGACCATGTTGTGGT |
|  | R: TCTAAGGCCTCAAAGCACCC |
| Unspecific monooxygenase (EC:1.14.14.1) | F: GAGCATGTCCTGGAGAAGGC |
|  | R: AAACCTTGTTGACGACGGGA |
