# Supplementary figures and images for: Next-generation sequencing of representational difference analysis products for identification of genes involved in diosgenin biosynthesis in fenugreek (Trigonella foenum-graecum)
Source: Planta. 2017 Feb 4;245(5):977–91. doi: 10.1007/s00425-017-2657-0 (PMC5393294; doi:10.1007/s00425-017-2657-0)

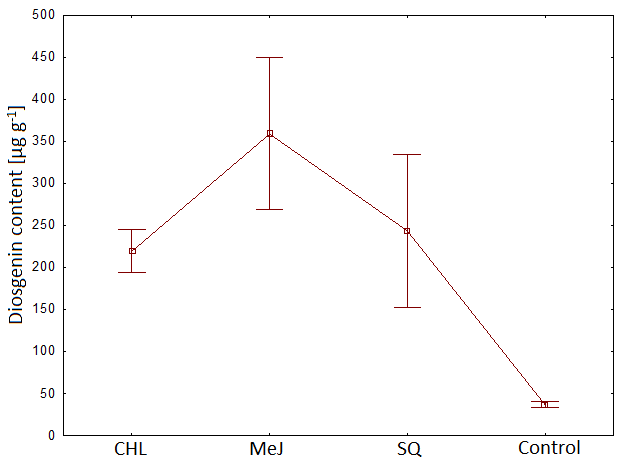

Supplement: Supplementary file 2 — Fig. S1 Average content of diosgenin in fenugreek plants (PNG 12 kb) [file 425_2017_2657_MOESM2_ESM.png]
