# Supplementary material for: Next-generation sequencing of representational difference analysis products for identification of genes involved in diosgenin biosynthesis in fenugreek (Trigonella foenum-graecum)
Source: Planta. 2017 Feb 4;245(5):977–91. doi: 10.1007/s00425-017-2657-0 (PMC5393294; doi:10.1007/s00425-017-2657-0)
Supplement: Supplementary file 3 — Supplementary material 3 (DOCX 12 kb) [file 425_2017_2657_MOESM3_ESM.docx]

Next generation sequencing of representational difference analysis products for identification of genes involved in diosgenin biosynthesis in fenugreek (*Trigonella foenum-graecum*), Planta, Ciura J, Szeliga M, Grzesik M, Tyrka M; Department of Biotechnology and Bioinformatics, Rzeszow University of Technology, Poland, mtyrka@prz.edu.pl

Table S2 Top-hit Blast results for species distribution

| Species | Number of sequences | | |
| --- | --- | --- | --- |
|  | RDA-CHL | RDA-MeJ | RDA-SQ |
| *Medicago truncatula* | 5,632 | 4,270 | 5,298 |
| *Cicer arietinum* | 2,405 | 1,719 | 2,233 |
| *Glycine max* | 472 | 343 | 432 |
| *Phaseolus vulgaris* | 208 | 159 | 207 |
