# Supplementary material for: Next-generation sequencing of representational difference analysis products for identification of genes involved in diosgenin biosynthesis in fenugreek (Trigonella foenum-graecum)
Source: Planta. 2017 Feb 4;245(5):977–91. doi: 10.1007/s00425-017-2657-0 (PMC5393294; doi:10.1007/s00425-017-2657-0)
Supplement: Supplementary file 4 — Supplementary material 4 (DOCX 13 kb) [file 425_2017_2657_MOESM4_ESM.docx]

Next generation sequencing of representational difference analysis products for identification of genes involved in diosgenin biosynthesis in fenugreek (*Trigonella foenum-graecum*), Planta, Ciura J, Szeliga M, Grzesik M, Tyrka M; Department of Biotechnology and Bioinformatics, Rzeszow University of Technology, Poland, mtyrka@prz.edu.pl

Table S3 Presentation of secondary metabolism at different GO levels for biological process

| GO terms | GO level | Number of sequences | | |
| --- | --- | --- | --- | --- |
|  |  | RDA-CHL | RDA-MeJ | RDA-SQ |
| GO:0009628 response to abiotic stimulus | 3 | 749 | 584 | 698 |
| GO:0009058 biosynthetic process | 3 | 2891 | 2375 | 2752 |
| GO:0044281 small molecule metabolic process | 4 | 2201 | 1830 | 2031 |
| GO:0019748 secondary metabolic process | 4 | 245 | 205 | 223 |
| GO:1901360 organic cyclic compound metabolic process | 4 | 2442 | 1963 | 2349 |
| GO:0008202 steroid metabolic process | 5 | 99 | 95 | 107 |
| GO:0009889 regulation of biosynthetic process | 5 | 879 | 700 | 855 |
| GO:0044550 secondary metabolite biosynthetic process | 5 | 141 | 126 | 134 |
| GO:0016125 sterol metabolic process | 6 | 44 | 48 | 51 |
| GO:0006694 steroid biosynthetic process | 6 | 94 | 90 | 104 |
| GO:0006720 isoprenoid metabolic process | 6 | 248 | 206 | 223 |
| GO:0016128 phytosteroid metabolic process | 6 | 20 | 28 | 31 |
| GO:0019216 regulation of lipid metabolic process | 6 | 24 | 16 | 21 |
| GO:0016126 sterol biosynthetic process | 7 | 42 | 44 | 49 |
| GO:0008299 isoprenoid biosynthetic process | 7 | 231 | 191 | 205 |
| GO:0006721 terpenoid metabolic process | 7 | 114 | 90 | 104 |
| GO:0016129 phytosteroid biosynthetic process | 7 | 20 | 28 | 31 |
| GO:0016114 terpenoid biosynthetic process | 8 | 108 | 84 | 98 |
