# Supplementary material for: Next-generation sequencing of representational difference analysis products for identification of genes involved in diosgenin biosynthesis in fenugreek (Trigonella foenum-graecum)
Source: Planta. 2017 Feb 4;245(5):977–91. doi: 10.1007/s00425-017-2657-0 (PMC5393294; doi:10.1007/s00425-017-2657-0)
Supplement: Supplementary file 5 — Supplementary material 5 (DOCX 26 kb) [file 425_2017_2657_MOESM5_ESM.docx]

Next generation sequencing of representational difference analysis products for identification of genes involved in diosgenin biosynthesis in fenugreek (*Trigonella foenum-graecum*), Planta, Ciura J, Szeliga M, Grzesik M, Tyrka M; Department of Biotechnology and Bioinformatics, Rzeszow University of Technology, Poland, mtyrka@prz.edu.pl

Table S4 Unigenes assigned to 3 levels in pathways analyzed by KEGG

| Categories  (Level 1) | Sub-categories  (Level 2) | Pathways  (Level 3) | Number of unigenes | | |
| --- | --- | --- | --- | --- | --- |
|  |  |  | RDA-CHL | RDA-MeJ | RDA-SQ |
| Metabolism | Carbohydrate metabolism | Glycolysis / Gluconeogenesis | 78 | 58 | 60 |
|  |  | Citrate cycle (TCA cycle) | 37 | 33 | 36 |
|  |  | Pentose phosphate pathway | 40 | 29 | 34 |
|  |  | Pentose and glucuronate interconversions | 28 | 19 | 24 |
|  |  | Fructose and mannose metabolism | 30 | 22 | 25 |
|  |  | Galactose metabolism | 36 | 24 | 31 |
|  |  | Ascorbate and aldarate metabolism | 17 | 14 | 18 |
|  |  | Starch and sucrose metabolism | 80 | 51 | 73 |
|  |  | Amino sugar and nucleotide sugar metabolism | 57 | 43 | 48 |
|  |  | Pyruvate metabolism | 55 | 57 | 52 |
|  |  | Glyoxylate and dicarboxylate metabolism | 36 | 43 | 33 |
|  |  | Propanoate metabolism | 16 | 20 | 17 |
|  |  | Butanoate metabolism | 11 | 7 | 8 |
|  |  | C5-Branched dibasic acid metabolism | 8 | 6 | 8 |
|  |  | Inositol phosphate metabolism | 27 | 20 | 27 |
|  | Energy metabolism | Oxidative phosphorylation | 74 | 60 | 63 |
|  |  | Photosynthesis | 38 | 32 | 36 |
|  |  | Photosynthesis - antenna proteins | 14 | 14 | 17 |
|  |  | Carbon fixation in photosynthetic organisms | 37 | 36 | 36 |
|  |  | Nitrogen metabolism | 13 | 14 | 14 |
|  |  | Sulfur metabolism | 18 | 16 | 20 |
|  | Lipid metabolism | Fatty acid biosynthesis | 16 | 16 | 19 |
|  |  | Fatty acid elongation | 5 | 6 | 7 |
|  |  | Fatty acid degradation | 23 | 23 | 20 |
|  |  | Cutin, suberine and wax biosynthesis | 6 | 8 | 7 |
|  |  | Steroid biosynthesis | 15 | 16 | 21 |
|  |  | Steroid hormone biosynthesis | 2 | 2 | 2 |
|  |  | Glycerolipid metabolism | 27 | 24 | 30 |
|  |  | Glycerophospholipid metabolism | 37 | 35 | 45 |
|  |  | Ether lipid metabolism | 12 | 11 | 14 |
|  |  | Sphingolipid metabolism | 10 | 14 | 13 |
|  |  | Arachidonic acid metabolism | 4 | 5 | 6 |
|  |  | Linoleic acid metabolism | 7 | 8 | 7 |
|  |  | alpha-Linolenic acid metabolism | 20 | 20 | 16 |
|  |  | Biosynthesis of unsaturated fatty acids | 16 | 14 | 12 |
|  | Nucleotide metabolism | Purine metabolism | 77 | 57 | 67 |
|  |  | Pyrimidine metabolism | 51 | 38 | 50 |
|  | Amino acid metabolism | Alanine, aspartate and glutamate metabolism | 27 | 30 | 28 |
|  |  | Glycine, serine and threonine metabolism | 42 | 43 | 37 |
|  |  | Cysteine and methionine metabolism | 42 | 37 | 41 |
|  |  | Valine, leucine and isoleucine degradation | 20 | 21 | 21 |
|  |  | Valine, leucine and isoleucine biosynthesis | 15 | 12 | 14 |
|  |  | Lysine biosynthesis | 8 | 7 | 7 |
|  |  | Lysine degradation | 16 | 13 | 12 |
|  |  | Arginine biosynthesis | 22 | 20 | 20 |
|  |  | Arginine and proline metabolism | 27 | 21 | 26 |
|  |  | Histidine metabolism | 10 | 9 | 10 |
|  |  | Tyrosine metabolism | 24 | 22 | 21 |
|  |  | Phenylalanine metabolism | 26 | 21 | 19 |
|  |  | Tryptophan metabolism | 12 | 9 | 8 |
|  |  | Phenylalanine, tyrosine and tryptophan biosynthesis | 26 | 19 | 23 |
|  | Metabolism of other amino acids | beta-Alanine metabolism | 23 | 15 | 21 |
|  |  | Taurine and hypotaurine metabolism | 8 | 5 | 8 |
|  |  | Phosphonate and phosphinate metabolism | 3 | 4 | 3 |
|  |  | Selenocompound metabolism | 8 | 11 | 12 |
|  |  | Cyanoamino acid metabolism | 21 | 15 | 19 |
|  |  | D-Glutamine and D-glutamate metabolism | 2 | 1 | 2 |
|  |  | Glutathione metabolism | 36 | 34 | 27 |
|  | Glycan biosynthesis and metabolism | N-Glycan biosynthesis | 22 | 15 | 16 |
|  |  | Various types of N-glycan biosynthesis | 17 | 11 | 13 |
|  |  | Other types of O-glycan biosynthesis | 4 | 3 | 3 |
|  |  | Glycosaminoglycan degradation | 7 | 7 | 4 |
|  |  | Glycosylphosphatidylinositol(GPI)-anchor biosynthesis | 4 | 2 | 1 |
|  |  | Glycosphingolipid biosynthesis - globo series | 2 | 5 | 3 |
|  |  | Glycosphingolipid biosynthesis - ganglio series | 2 | 2 | 2 |
|  |  | Lipopolysaccharide biosynthesis | 4 | 0 | 3 |
|  |  | Other glycan degradation | 9 | 8 | 10 |
|  | Metabolism of cofactors and vitamins | Thiamine metabolism | 9 | 4 | 5 |
|  |  | Riboflavin metabolism | 3 | 2 | 4 |
|  |  | Vitamin B6 metabolism | 2 | 2 | 4 |
|  |  | Nicotinate and nicotinamide metabolism | 13 | 6 | 12 |
|  |  | Pantothenate and CoA biosynthesis | 14 | 10 | 13 |
|  |  | Biotin metabolism | 6 | 5 | 5 |
|  |  | Lipoic acid metabolism | 4 | 2 | 2 |
|  |  | Folate biosynthesis | 6 | 4 | 7 |
|  |  | One carbon pool by folate | 12 | 11 | 12 |
|  |  | Retinol metabolism | 7 | 6 | 4 |
|  |  | Porphyrin and chlorophyll metabolism | 31 | 24 | 23 |
|  |  | Ubiquinone and other terpenoid-quinone biosynthesis | 17 | 16 | 16 |
|  | Metabolism of terpenoids and polyketides | Terpenoid backbone biosynthesis | 21 | 19 | 22 |
|  |  | Sesquiterpenoid and triterpenoid biosynthesis | 7 | 7 | 6 |
|  |  | Diterpenoid biosynthesis | 3 | 3 | 2 |
|  |  | Carotenoid biosynthesis | 16 | 11 | 15 |
|  |  | Brassinosteroid biosynthesis | 4 | 2 | 2 |
|  |  | Insect hormone biosynthesis | 1 | 1 | 0 |
|  |  | Zeatin biosynthesis | 4 | 1 | 3 |
|  |  | Limonene and pinene degradation | 3 | 5 | 4 |
|  |  | Biosynthesis of ansamycins | 2 | 2 | 3 |
|  |  | Tetracycline biosynthesis | 2 | 3 | 3 |
|  |  | Polyketide sugar unit biosynthesis | 1 | 1 | 1 |
|  | Biosynthesis of other secondary metabolites | Phenylpropanoid biosynthesis | 52 | 35 | 46 |
|  |  | Stilbenoid, diarylheptanoid and gingerol biosynthesis | 6 | 9 | 8 |
|  |  | Flavonoid biosynthesis | 13 | 16 | 16 |
|  |  | Flavone and flavonol biosynthesis | 3 | 2 | 2 |
|  |  | Anthocyanin biosynthesis | 1 | 1 | 1 |
|  |  | Isoflavonoid biosynthesis | 7 | 4 | 6 |
|  |  | Isoquinoline alkaloid biosynthesis | 13 | 11 | 12 |
|  |  | Tropane, piperidine and pyridine alkaloid biosynthesis | 14 | 12 | 11 |
|  |  | Caffeine metabolism | 1 | 1 | 1 |
|  |  | Betalain biosynthesis | 2 | 1 | 1 |
|  |  | Glucosinolate biosynthesis | 2 | 0 | 1 |
|  |  | Monobactam biosynthesis | 5 | 5 | 5 |
|  |  | Streptomycin biosynthesis | 11 | 5 | 8 |
|  |  | Butirosin and neomycin biosynthesis | 5 | 1 | 2 |
|  |  | Novobiocin biosynthesis | 3 | 3 | 3 |
|  |  | Aflatoxin biosynthesis | 2 | 1 | 2 |
|  | Xenobiotics biodegradation and metabolism | Aminobenzoate degradation | 4 | 6 | 3 |
|  |  | Chloroalkane and chloroalkene degradation | 8 | 6 | 7 |
|  |  | Styrene degradation | 4 | 3 | 3 |
|  |  | Naphthalene degradation | 5 | 4 | 4 |
|  |  | Metabolism of xenobiotics by cytochrome P450 | 17 | 17 | 12 |
|  |  | Drug metabolism - cytochrome P450 | 17 | 17 | 12 |
|  |  | Drug metabolism - other enzymes | 6 | 3 | 6 |
| Genetic information processing | Transcription | RNA polymerase | 23 | 19 | 26 |
|  |  | Basal transcription factors | 16 | 8 | 15 |
|  |  | Spliceosome | 84 | 71 | 92 |
|  | Translation | Ribosome | 100 | 92 | 102 |
|  |  | Aminoacyl-tRNA biosynthesis | 39 | 32 | 33 |
|  |  | RNA transport | 88 | 65 | 81 |
|  |  | mRNA surveillance pathway | 60 | 45 | 52 |
|  |  | Ribosome biogenesis in eukaryotes | 38 | 38 | 40 |
|  | Folding, sorting and degradation | Protein export | 19 | 13 | 19 |
|  |  | Protein processing in endoplasmic reticulum | 90 | 75 | 78 |
|  |  | SNARE interactions in vesicular transport | 16 | 12 | 16 |
|  |  | Ubiquitin mediated proteolysis | 53 | 39 | 52 |
|  |  | Sulfur relay system | 5 | 4 | 5 |
|  |  | Proteasome | 40 | 39 | 39 |
|  |  | RNA degradation | 42 | 38 | 36 |
|  | Replication and repair | DNA replication | 11 | 8 | 6 |
|  |  | Base excision repair | 12 | 8 | 9 |
|  |  | Nucleotide excision repair | 20 | 12 | 14 |
|  |  | Mismatch repair | 7 | 5 | 7 |
|  |  | Homologous recombination | 12 | 6 | 16 |
|  |  | Non-homologous end-joining | 3 | 1 | 4 |
|  |  | Fanconi anemia pathway | 11 | 7 | 10 |
| Environmental information processing | Membrane transport | ABC transporters | 5 | 4 | 8 |
|  |  | Bacterial secretion system | 9 | 5 | 10 |
|  | Signal transduction | Two-component system | 10 | 12 | 13 |
|  |  | Ras signaling pathway | 16 | 14 | 14 |
|  |  | Rap1 signaling pathway | 7 | 6 | 6 |
|  |  | MAPK signaling pathway | 15 | 12 | 15 |
|  |  | ErbB signaling pathway | 9 | 5 | 8 |
|  |  | Wnt signaling pathway | 19 | 15 | 17 |
|  |  | Notch signaling pathway | 5 | 3 | 3 |
|  |  | Hedgehog signaling pathway | 3 | 2 | 2 |
|  |  | TGF-beta signaling pathway | 17 | 12 | 12 |
|  |  | Hippo signaling pathway | 14 | 11 | 12 |
|  |  | VEGF signaling pathway | 7 | 5 | 6 |
|  |  | NF-kappa B signaling pathway | 6 | 5 | 5 |
|  |  | TNF signaling pathway | 6 | 4 | 5 |
|  |  | HIF-1 signaling pathway | 29 | 17 | 23 |
|  |  | FoxO signaling pathway | 29 | 19 | 20 |
|  |  | Calcium signaling pathway | 8 | 9 | 8 |
|  |  | Phosphatidylinositol signaling system | 23 | 17 | 23 |
|  |  | Sphingolipid signaling pathway | 29 | 26 | 25 |
|  |  | cAMP signaling pathway | 22 | 16 | 17 |
|  |  | cGMP - PKG signaling pathway | 15 | 14 | 14 |
|  |  | PI3K-Akt signaling pathway | 40 | 32 | 38 |
|  |  | AMPK signaling pathway | 46 | 32 | 41 |
|  |  | mTOR signaling pathway | 21 | 16 | 20 |
|  |  | Plant hormone signal transduction | 59 | 62 | 66 |
| **Cellular Processes** | Transport and catabolism | Endocytosis | 60 | 48 | 61 |
|  |  | Phagosome | 37 | 29 | 32 |
|  |  | Lysosome | 44 | 31 | 34 |
|  |  | Peroxisome | 35 | 33 | 34 |
|  |  | Regulation of autophagy | 14 | 11 | 11 |
| **Organismal Systems** | Immune system | Cytosolic DNA-sensing pathway | 10 | 9 | 12 |
|  |  | Natural killer cell mediated cytotoxicity | 7 | 5 | 6 |
|  | Environmental adaptation | Circadian rhythm | 14 | 8 | 6 |
|  |  | Circadian rhythm - plant | 21 | 17 | 18 |
|  |  | Plant-pathogen interaction | 57 | 53 | 56 |
