# Supplementary material for: Next-generation sequencing of representational difference analysis products for identification of genes involved in diosgenin biosynthesis in fenugreek (Trigonella foenum-graecum)
Source: Planta. 2017 Feb 4;245(5):977–91. doi: 10.1007/s00425-017-2657-0 (PMC5393294; doi:10.1007/s00425-017-2657-0)
Supplement: Supplementary file 6 — Supplementary material 6 (DOCX 14 kb) [file 425_2017_2657_MOESM6_ESM.docx]

Next generation sequencing of representational difference analysis products for identification of genes involved in diosgenin biosynthesis in fenugreek (*Trigonella foenum-graecum*), Planta, Ciura J, Szeliga M, Grzesik M, Tyrka M; Department of Biotechnology and Bioinformatics, Rzeszow University of Technology, Poland, mtyrka@prz.edu.pl

Table S5 KEGG pathways related to secondary metabolism

| Secondary metabolites biosynthesis pathways | Number of unigenes | | |
| --- | --- | --- | --- |
|  | RDA-CHL | RDA-MeJ | RDA-SQ |
| Anthocyanin biosynthesis [PATH:ko00942] | 1 | 1 | 1 |
| Betalain biosynthesis [PATH:ko00965] | 2 | 1 | 1 |
| Biosynthesis of ansamycins [PATH:ko01051] | 2 | 2 | 3 |
| Brassinosteroid biosynthesis [PATH:ko00905] | 4 | 2 | 2 |
| Caffeine metabolism [PATH:ko00232] | 1 | 1 | 1 |
| Carotenoid biosynthesis [PATH:ko00906] | 16 | 11 | 15 |
| Cutin, suberine and wax biosynthesis [PATH:ko00073] | 6 | 8 | 7 |
| Geraniol degradation [PATH:ko00281] | 0 | 1 | 1 |
| Glucosinolate biosynthesis [PATH:ko00966] | 2 | 0 | 1 |
| Diterpenoid biosynthesis [PATH:ko00904] | 3 | 3 | 2 |
| Flavone and flavonol biosynthesis [PATH:ko00944] | 3 | 2 | 2 |
| Flavonoid biosynthesis [PATH:ko00941] | 13 | 16 | 16 |
| Insect hormone biosynthesis [PATH:ko00981] | 1 | 1 | 0 |
| Isoflavonoid biosynthesis [PATH:ko00943] | 7 | 4 | 6 |
| Isoquinoline alkaloid biosynthesis [PATH:ko00950] | 13 | 11 | 12 |
| Limonene and pinene degradation [PATH:ko00903] | 3 | 5 | 4 |
| Monoterpenoid biosynthesis [PATH:ko00902] | 1 | 0 | 0 |
| Phenylpropanoid biosynthesis [PATH:ko00940] | 52 | 35 | 46 |
| Sesquiterpenoid and triterpenoid biosynthesis [PATH:ko00909] | 7 | 7 | 6 |
| Steroid biosynthesis [PATH:ko00100] | 15 | 16 | 21 |
| Stilbenoid, diarylheptanoid and gingerol biosynthesis [PATH:ko00945] | 6 | 9 | 8 |
| Terpenoid backbone biosynthesis [PATH:ko00900] | 21 | 19 | 22 |
| Tetracycline biosynthesis [PATH:ko00253] | 2 | 3 | 3 |
| Tropane, piperidine and pyridine alkaloid biosynthesis [PATH:ko00960] | 14 | 12 | 11 |
| Ubiquinone and other terpenoid-quinone biosynthesis [PATH:ko00130] | 17 | 16 | 16 |
| Zeatin biosynthesis [PATH:ko00908] | 4 | 1 | 3 |
| Total | 216 | 187 | 210 |
