# Supplementary material for: Next-generation sequencing of representational difference analysis products for identification of genes involved in diosgenin biosynthesis in fenugreek (Trigonella foenum-graecum)
Source: Planta. 2017 Feb 4;245(5):977–91. doi: 10.1007/s00425-017-2657-0 (PMC5393294; doi:10.1007/s00425-017-2657-0)
Supplement: Supplementary file 7 — Supplementary material 7 (DOCX 14 kb) [file 425_2017_2657_MOESM7_ESM.docx]

Next generation sequencing of representational difference analysis products for identification of genes involved in diosgenin biosynthesis in fenugreek (*Trigonella foenum-graecum*), Planta, Ciura J, Szeliga M, Grzesik M, Tyrka M; Department of Biotechnology and Bioinformatics, Rzeszow University of Technology, Poland, mtyrka@prz.edu.pl

Table S6 Summary of assigned unigenes to 25 clusters of orthologous groups (KOG)

| KOG class | KOG categories | Number of unigenes | | |
| --- | --- | --- | --- | --- |
|  |  | RDA-CHL | RDA-MeJ | RDA-SQ |
| A | RNA processing and modification | 274 | 226 | 269 |
| B | Chromatin structure and dynamics | 82 | 50 | 77 |
| C | Energy production and conversion | 327 | 310 | 301 |
| D | Cell cycle control, cell division, chromosome partitioning | 120 | 101 | 112 |
| E | Amino acid transport and metabolism | 337 | 293 | 354 |
| F | Nucleotide transport and metabolism | 75 | 51 | 58 |
| G | Carbohydrate transport and metabolism | 438 | 352 | 410 |
| H | Coenzyme transport and metabolism | 98 | 80 | 87 |
| I | Lipid transport and metabolism | 250 | 227 | 249 |
| J | Translation, ribosomal structure and biogenesis | 451 | 400 | 418 |
| K | Transcription | 301 | 259 | 295 |
| L | Replication, recombination and repair | 108 | 77 | 97 |
| M | Cell wall/membrane/envelope biogenesis | 91 | 64 | 86 |
| N | Cell motility | 2 | 1 | 0 |
| O | Posttranslational modification, protein turnover, chaperones | 698 | 613 | 704 |
| P | Inorganic ion transport and metabolism | 150 | 120 | 148 |
| Q | Secondary metabolites biosynthesis, transport and catabolism | 261 | 211 | 234 |
| R | General function prediction only | 697 | 562 | 667 |
| S | Function unknown | 262 | 177 | 245 |
| T | Signal transduction mechanisms | 717 | 545 | 659 |
| U | Intracellular trafficking, secretion, and vesicular transport | 330 | 258 | 299 |
| V | Defense mechanisms | 47 | 37 | 44 |
| W | Extracellular structures | 15 | 9 | 13 |
| Y | Nuclear structure | 35 | 21 | 25 |
| Z | Cytoskeleton | 158 | 152 | 154 |
