# Supplementary material for: Next-generation sequencing of representational difference analysis products for identification of genes involved in diosgenin biosynthesis in fenugreek (Trigonella foenum-graecum)
Source: Planta. 2017 Feb 4;245(5):977–91. doi: 10.1007/s00425-017-2657-0 (PMC5393294; doi:10.1007/s00425-017-2657-0)
Supplement: Supplementary file 8 — Supplementary material 8 (DOCX 13 kb) [file 425_2017_2657_MOESM8_ESM.docx]

Next generation sequencing of representational difference analysis products for identification of genes involved in diosgenin biosynthesis in fenugreek (*Trigonella foenum-graecum*), Planta, Ciura J, Szeliga M, Grzesik M, Tyrka M; Department of Biotechnology and Bioinformatics, Rzeszow University of Technology, Poland, mtyrka@prz.edu.pl

Table S7 The 15 most frequently occurring Pfam domains/families in fenugreek transcripts

| Rank | Conserved domain/family | Accesion ID | Number of sequences | | |
| --- | --- | --- | --- | --- | --- |
|  |  |  | RDA-CHL | RDA-MeJ | RDA-SQ |
| 1 | Protein kinase domain | PF00069.20 | 267 | 177 | 220 |
| 2 | Protein tyrosine kinase | PF07714.12 | 246 | 160 | 201 |
| 3 | WD domain, G-beta repeat | PF00400.27 | 94 | 72 | 82 |
| 4 | RNA recognition motif. (a.k.a. RRM, RBD, or RNP domain) | PF00076.17 | 78 | 71 | 77 |
| 5 |  | PF14259.1 | 69 | 66 | 68 |
| 6 |  | PF13893.1 | 66 | 60 | 66 |
| 7 | Major Facilitator Superfamily | PF07690.11 | 84 | 60 | 78 |
| 8 | Ring finger domain | PF13639.1 | 80 | 55 | 79 |
| 9 | Zinc finger, C3HC4 type (RING finger) | PF13923.1 | 78 | 57 | 73 |
| 10 |  | PF13920.1 | 73 | 58 | 75 |
| 11 |  | PF00097.20 | 67 | 49 | 62 |
| 12 | Cytochrome P450 | PF00067.17 | 78 | 62 | 65 |
| 13 | IncA protein | PF04156.9 | 72 | 47 | 67 |
| 14 | Leucine Rich repeats (2 copies) | PF12799.2 | 70 | 50 | 62 |
| 15 | Growth-arrest specific micro-tubule binding | PF13851.1 | 58 | 48 | 58 |
