# Supplementary material for: Next-generation sequencing of representational difference analysis products for identification of genes involved in diosgenin biosynthesis in fenugreek (Trigonella foenum-graecum)
Source: Planta. 2017 Feb 4;245(5):977–91. doi: 10.1007/s00425-017-2657-0 (PMC5393294; doi:10.1007/s00425-017-2657-0)
Supplement: Supplementary file 9 — Supplementary material 9 (DOCX 17 kb) [file 425_2017_2657_MOESM9_ESM.docx]

Next generation sequencing of representational difference analysis products for identification of genes involved in diosgenin biosynthesis in fenugreek (*Trigonella foenum-graecum*), Planta, Ciura J, Szeliga M, Grzesik M, Tyrka M; Department of Biotechnology and Bioinformatics, Rzeszow University of Technology, Poland, mtyrka@prz.edu.pl

Table S8 Enzymes involved in terpenoid and steroid biosynthetic pathway. Enzymes were grouped into mevalonate pathway, methylerythritol 4-phosphate (MEP) pathway, squalene and sterols biosynthesis

| Enzyme name | EC number | Enzyme | Number of unigenes | | |
| --- | --- | --- | --- | --- | --- |
|  |  | abbreviation | RDA-CHL | RDA-MeJ | RDA-SQ |
| Isopentenyl-PP biosynthesis, mevalonate pathway |  |  |  |  |  |
| Acetyl-CoA acetyltransferase | 2.3.1.9 | AACT | 0 | 2 | 0 |
| Hydroxymethylglutaryl-CoA synthase | 2.3.3.10 | HMGS | 0 | 0 | 0 |
| Hydroxymethylglutaryl-CoA reductase | 1.1.1.34 | HMGCR | 4 | 5 | 3 |
| Mevalonate kinase | 2.7.1.36 | MVK | 2 | 1 | 1 |
| Phosphomevalonate kinase | 2.7.4.2 | PMK | 1 | 0 | 1 |
| Diphosphomevalonate decarboxylase | 4.1.1.33 | MVD | 2 | 1 | 2 |
| Isopentenyl-diphosphate delta-isomerase | 5.3.3.2 | IPI | 0 | 0 | 0 |
| Isopentenyl-PP biosynthesis, MEP pathway |  |  |  |  |  |
| 1-deoxy-D-xylulose-5-phosphate synthase | 2.2.1.7 | DXS | 1 | 1 | 1 |
| 1-deoxy-D-xylulose-5-phosphate reductoisomerase | 1.1.1.267 | DXR | 2 | 3 | 2 |
| 2-C-methyl-D-erythritol 4-phosphate cytidylyltransferase | 2.7.7.60 | CDP-MES | 0 | 0 | 0 |
| 4-diphosphocytidyl-2-C-methyl-D-erythritol kinase | 2.7.1.148 | CDP-MEK (ispE) | 2 | 2 | 2 |
| 2-C-methyl-D-erythritol 2,4-cyclodiphosphate synthase | 4.6.1.12 | MECPS (ispF) | 1 | 1 | 1 |
| (E)-4-hydroxy-3-methylbut-2-enyl-diphosphate synthase | 1.17.7.1 | HDS (gcpE) | 3 | 0 | 4 |
| 4-hydroxy-3-methylbut-2-enyl diphosphate reductase | 1.17.1.2 | HDR (ispH) | 1 | 1 | 1 |
| Squalene biosynthesis |  |  |  |  |  |
| Geranyl diphosphate synthase | 2.5.1.1 | GPS | 6 | 3 | 6 |
| (2E,6E)-Farnesyl diphosphate synthase | 2.5.1.10 | FDPS | 3 | 1 | 1 |
| Geranylgeranyl diphosphate synthase | 2.5.1.29 | GGPS | 3 | 1 | 3 |
| Squalene synthase/farnesyl-diphosphate farnesyltransferase | 2.5.1.21 | SQS | 2 | 1 | 2 |
| Squalene monooxygenase/squalene epoxidase | 1.14.13.132 | SQE | 1 | 2 | 1 |
| Biosynthesis of sterols (i.e. diosgenin) |  |  |  |  |  |
| Cycloartenol synthase | 5.4.99.8 | CAS | 1 | 1 | 0 |
| Sterol 24-C-methyltransferase | 2.1.1.41 | SMT1 | 1 | 1 | 3 |
| Sterol-4alpha-methyl oxidase | 1.14.13.72 | SMO | 2 | 1 | 1 |
| 24-methylenesterol C-methyltransferase | 2.1.1.143 | SMT2 | 1 | 1 | 1 |
| Cycloeucalenol cycloisomerase/cyclopropyl isomerase | 5.5.1.9 | CPI | 1 | 1 | 2 |
| Cytochrome P450, family 51 (sterol 14-demethylase) | 1.14.13.70 | CYP51 | 2 | 2 | 2 |
| Delta14-sterol reductase | 1.3.1.70 | FK | 2 | 1 | 1 |
| Delta(8)-delta(7) isomerase | 5.3.3.5 | HYD1 | 0 | 1 | 1 |
| Delta(7)-sterol-C5(6)-desaturase/lathosterol oxidase | 1.14.21.6 | DWF7 | 0 | 1 | 0 |
| 7-dehydrocholesterol reductase | 1.3.1.21 | DWF5 | 2 | 3 | 3 |
| Delta(24)-sterol reductase/lanosterol delta(24)-reductase | 1.3.1.72 | DWF1 | 3 | 3 | 3 |
| Lanosterol synthase | 5.4.99.7 | LAS | 0 | 0 | 0 |
| 3-beta-hydroxysteroid-4-alpha-carboxylate 3-dehydrogenase | 1.1.1.170 | HSD | 3 | 4 | 3 |
| 3-beta-hydroxysteroid 3-dehydrogenase/3-keto-steroid reductase | 1.1.1.270 | KR | 0 | 0 | 0 |
| TOTAL |  |  | 52 | 45 | 51 |
